# Crossed-Beam and Theoretical Studies of the O(^3^P, ^1^D) + Benzene Reactions: Primary Products, Branching Fractions, and Role of Intersystem Crossing

**DOI:** 10.1021/acs.jpca.1c06913

**Published:** 2021-09-17

**Authors:** Gianmarco Vanuzzo, Adriana Caracciolo, Timothy K. Minton, Nadia Balucani, Piergiorgio Casavecchia, Carlo de Falco, Alberto Baggioli, Carlo Cavallotti

**Affiliations:** † Dipartimento di Chimica, Biologia e Biotecnologie, Università degli Studi di Perugia, 06123 Perugia, Italy; ‡ MOX − Modellistica e Calcolo Scientifico, Dipartimento di Matematica, Politecnico di Milano, 20133 Milano, Italy; § Dipartimento di Chimica, Materiali e Ingegneria Chimica, Politecnico di Milano, 20131 Milano, Italy

## Abstract

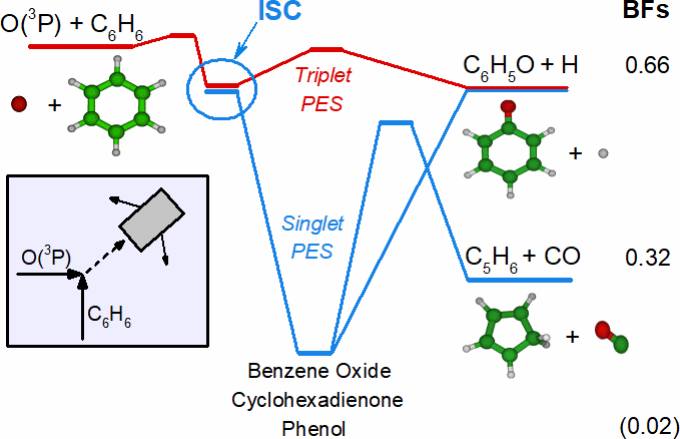

Reliable modeling of hydrocarbon oxidation relies critically on knowledge of the branching fractions (BFs) as a function of temperature (*T*) and pressure (*p*) for the products of the reaction of the hydrocarbon with atomic oxygen in its ground state, O(^3^P). During the past decade, we have performed in-depth investigations of the reactions of O(^3^P) with a variety of small unsaturated hydrocarbons using the crossed molecular beam (CMB) technique with *universal* mass spectrometric (MS) detection and time-of-flight (TOF) analysis, combined with synergistic theoretical calculations of the relevant potential energy surfaces (PESs) and statistical computations of product BFs, including intersystem crossing (ISC). This has allowed us to determine the primary products, their BFs, and extent of ISC to ultimately provide theoretical channel-specific rate constants as a function of *T* and *p*. In this work, we have extended this approach to the oxidation of one of the most important species involved in the combustion of aromatics: the benzene (C_6_H_6_) molecule. Despite extensive experimental and theoretical studies on the kinetics and dynamics of the O(^3^P) + C_6_H_6_ reaction, the relative importance of the C_6_H_5_O (phenoxy) + H open-shell products and of the spin-forbidden C_5_H_6_ (cyclopentadiene) + CO and phenol adduct closed-shell products are still open issues, which have hampered the development of reliable benzene combustion models. With the CMB technique, we have investigated the reaction dynamics of O(^3^P) + benzene at a collision energy (*E*_c_) of 8.2 kcal/mol, focusing on the occurrence of the phenoxy + H and spin-forbidden C_5_H_6_ + CO and phenol channels in order to shed further light on the dynamics of this complex and important reaction, including the role of ISC. Concurrently, we have also investigated the reaction dynamics of O(^1^D) + benzene at the same *E*_c_. Synergistic high-level electronic structure calculations of the underlying triplet/singlet PESs, including nonadiabatic couplings, have been performed to complement and assist the interpretation of the experimental results. Statistical (RRKM)/master equation (ME) computations of the product distribution and BFs on these PESs, with inclusion of ISC, have been performed and compared to experiment. In light of the reasonable agreement between the CMB experiment, literature kinetic experimental results, and theoretical predictions for the O(^3^P) + benzene reaction, the so-validated computational methodology has been used to predict (i) the BF between the C_6_H_5_O + H and C_5_H_6_ + CO channels as a function of collision energy and temperature (at 0.1 and 1 bar), showing that their increase progressively favors radical (phenoxy + H)-forming over molecule (C_5_H_6_ + CO and phenol stabilization)-forming channels, and (ii) channel-specific rate constants as a function of *T* and *p*, which are expected to be useful for improved combustion models.

## 1 Introduction

Since the early pioneering work of Cvetanovic in the 1950s,^1−3^ the reactions of ground-state atomic oxygen, O(^3^P), with unsaturated hydrocarbons (UHs) (alkynes, alkenes, dienes, and aromatics) have received a great deal of attention because of their importance in atmospheric chemistry^4^ and especially combustion chemistry.^5−8^ Initially, the effort was mainly devoted to kinetics,^3^ but, starting from the early 1980s, work on dynamics under single-collision conditions was also undertaken using a variety of techniques, ranging from crossed molecular beam (CMB) methods with mass spectrometric (MS) detection^9−14^ to laser-based spectroscopic techniques in a cell or flow system.^15,16^ However, the characterization of the detailed reaction mechanism, in particular the determination of the relative importance of the various competing reaction channels, has always been a challenge. As a consequence, results have often been fraught with uncertainty and controversy (see, e.g., Table 2 in ref (17)). It is worth recalling that the reactions of O(^3^P) with UHs are multichannel nonadiabatic reactions, in which intersystem crossing (ISC) from the entrance triplet potential energy surface (PES) to the underlying singlet PES plays a central role,^10−12,17−19^ and this makes the detailed characterization of the reaction dynamics quite taxing. In fact, detailed comprehension of the mechanism of the combustion-relevant multichannel reactions of O(^3^P) with UHs requires the identification of all primary reaction products, the determination of their branching fractions (BFs), and an assessment of the role of ISC. This can be achieved by combining CMB experiments (using universal soft electronionization MS detection and time-of-flight (TOF) analysis) with high-level ab initio electronic structure calculations of the triplet/singlet PESs and their couplings, and Rice–Ramsperger–Kassel–Marcus/master equation (RRKM/ME) computations of product BFs including ISC.^19−23^ We emphasize that reliable information on product BFs as a function of temperature and then predictions of channel-specific rate constants as a function of temperature and pressure are crucially needed to improve current combustion models.^19−24^

Over the past several years we have investigated in combined CMB/theoretical studies the dynamics of a variety of reactions of O(^3^P) with UHs (alkynes, alkenes, and dienes) involving two, three, and four carbon atoms, such as acetylene,^25^ ethylene,^17,26−28^ propyne,^21,29^ propene,^20,30^ allene (propadiene),^31^ 1-butene,^22^ 1,2-butadiene,^32^ and 1,3-butadiene.^33^ In particular, exploiting *soft* electron ionization, we have been able to identify for the first time all of the primary reaction product channels (up to six or seven for some of the above systems) and determine their BFs. The experimental BFs have usually been compared with RRKM/ME statistical predictions on state-of-the-art triplet/singlet PESs with inclusion of ISC. Once the statistical predictions were validated by experiment, theory was used to predict BFs and extent of ISC as a function of temperature and pressure,^22,23^ ultimately for inclusion in combustion models.

Very recently, we have extended our CMB investigations of reactions of O(^3^P) with UHs also to aromatic hydrocarbons (AHs) such as benzene, toluene, and the prototypical heteroaromatic pyridine. Preliminary results on the reaction of O(^3^P) with the exemplary aromatic molecule benzene were recently reported in the form of a Letter.^34^ As in most of our recent studies, the CMB investigation was accompanied by synergistic theoretical calculations of the triplet/singlet PESs as well as statistical calculations of BFs, with inclusion of ISC effects, to derive channel-specific rate constants as a function of temperature and pressure for inclusion in improved combustion models of the important O(^3^P) + benzene system. Here we report a full account of this study.

Among the AHs, benzene certainly plays a crucial role because it is a large component of fuels, is also commonly formed in combustion of aliphatics, and generates polycyclic aromatic hydrocarbons (PAHs) and soot,^6^ which significantly affect atmospheric chemistry, the environment, and also our health.^4,35,36^ For these reasons, during the past decades, benzene oxidation has been studied in detail in order to reach a global understanding of hydrocarbon combustion processes.^2,10,14,24,34,37−48^ However, up to the present days, the detailed dynamics (including primary products and BFs) of the O(^3^P) + benzene reaction was still not well-understood, which has hampered the development of detailed, reliable chemical kinetic combustion models.^7,49,50^ In fact, although many kinetic models have been proposed for benzene oxidation, their validity, as well as that of models for the combustion of other one-ring aromatics such as toluene and xylenes, are to a great extent subject to uncertainties in the identities of the products and the BFs of the reaction between benzene and O(^3^P).

Although numerous studies have in fact been carried out on the O(^3^P) + benzene reaction from both theoretical and experimental points of view, the extent of formation of the spin-forbidden CO + C_5_H_6_ (cyclopentadiene) products of this reaction, which implies an ISC process from the triplet PES to the underlying singlet PES, is still an open question. A detailed review of previous studies is provided in the Supporting Information (SI). To shed further light on, with the aim to clarify, the overall dynamics of the O(^3^P) + benzene system, including the role of ISC, we have investigated this reaction at a collision energy (*E*_c_) of 8.2 kcal/mol under single-collision conditions using the CMB technique with universal MS detection and TOF analysis. Notably, because we use a supersonic beam of atomic oxygen containing mainly ground-state O(^3^P) but also a significant fraction of excited O(^1^D) (see section 2), the detailed dynamics of the O(^1^D) + benzene reaction was also characterized at the same *E*_c_ and compared with that of the O(^3^P) reaction as well as with previous CMB results^51^ on O(^1^D) + C_6_H_6_.

On the basis of the theoretical part of the present work,^34^ which supersedes previous theoretical studies,^24,48^ the energetically available reactive channels for the O(^3^P, ^1^D) + benzene reactions are the following:
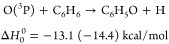
1
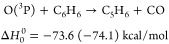
2
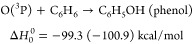
3
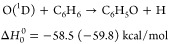
4
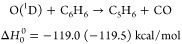
5
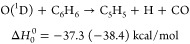
6The reported enthalpies of reaction at 0 K are those calculated in this work (the values in parentheses are experimental values from recommended Δ*H*_0_^0^);^52^ C_5_H_6_ stands for 1,3-cyclopentadiene and C_5_H_5_ for the cyclopentadienyl radical. It should be noted that the H abstraction channel leading to OH + C_6_H_5_ (phenyl) is endoergic by about 10 kcal/mol for O(^3^P) and exhibits a high energy barrier (of about 12 kcal/mol);^48^ therefore, it is expected to contribute negligibly to the O(^3^P) reaction and to be minor also for the O(^1^D) reaction^51^ (see the SI).

**Figure 1:**
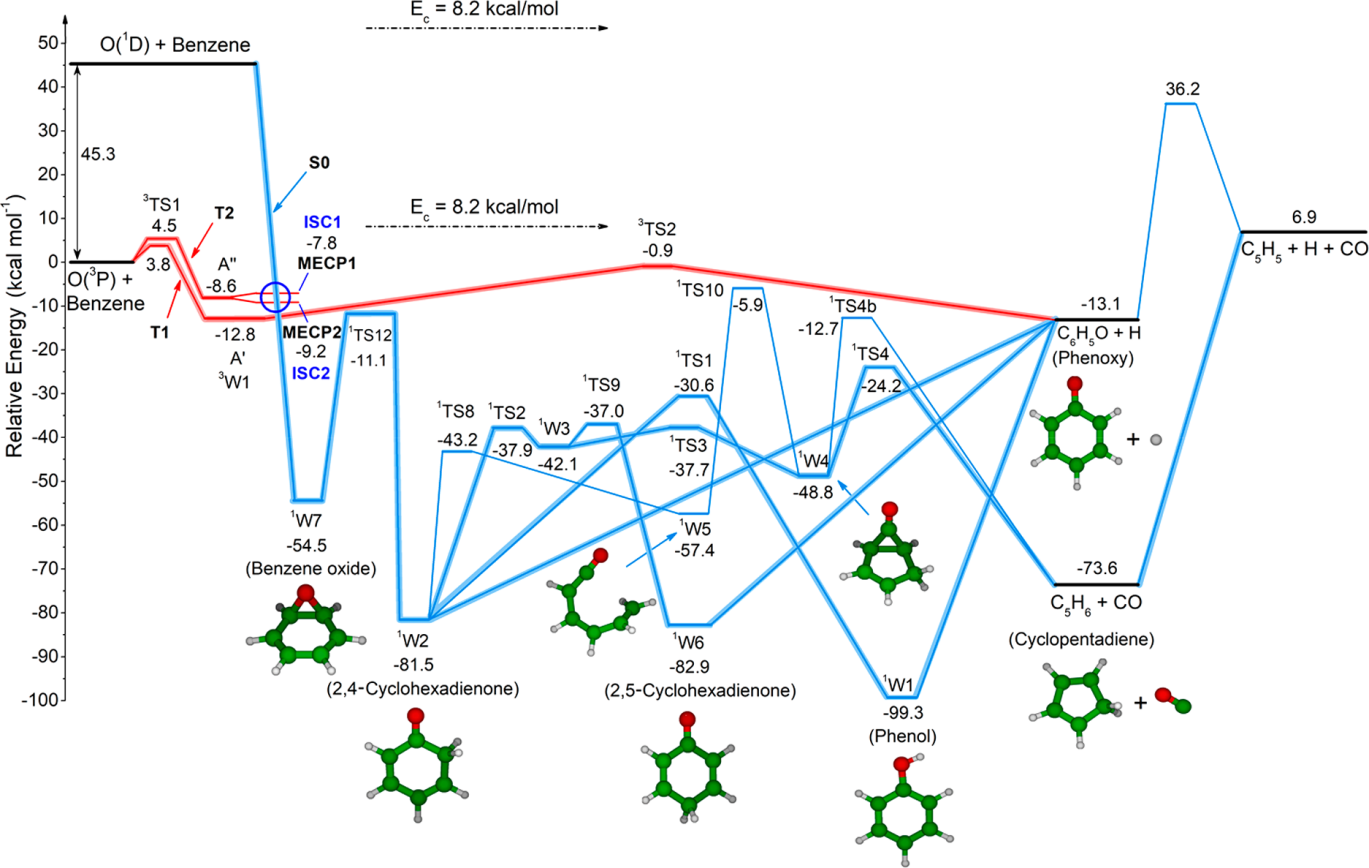
Triplet (T1, T2) (red lines) and singlet (S0) (blue lines) potential energy surfaces for the O(^3^P) + C_6_H_6_ reaction (kcal/mol) (also see ref (34)). Intersystem crossing (ISC) structures were determined at the ωB97X-D/6-311+G(d,p) level using unrestricted (ISC1) and restricted (ISC2) wave functions. The reactants and the main observed products (phenoxy + H, cyclopentadiene + CO, and cyclopentadienyl + H + CO, are indicated in black. The abstraction channel leading to OH + C_6_H_5_ (phenyl), which is endothermic for O(^3^P) by about 12 kcal/mol (see ref (48)) and exothermic for O(^1^D) by about 36 kcal/mol (see ref (51) and the SI), the pathway from ^3^W1(A″) to phenoxy + H, which has a high transition state energy of about 16 kcal/mol with respect to reactants (see ref (48)), and the pathway to C_6_H_4_(benzyne) + H_2_O products (Δ*H*_0_^0^ = −26.5 kcal/mol; see ref (34)), which have very low probabilities of formation, are not shown.

The reactivity that follows addition of O(^3^P) to benzene is well-described by the C_6_H_6_O potential energy surface shown in Figure 1 (see section 2.2 and ref (34)), which represents a very significant improvement with respect to previous PESs.^24,48^ In fact, as discussed in ref (34) and below, besides the multireference character of some important aspects of the triplet and singlet PESs, which required appropriate high-level quantum treatment to obtain more accurate energies, it is the detailed treatment of ISC that was missing for this system until the present study (see section 2.2).^34^ This turned out to be crucial for reliable treatment of the reaction kinetics and dynamics. In Figure 1 we have highlighted in red the adiabatic triplet pathways leading to phenoxy + H products and to the minimum-energy crossing points (MECPs) where ISC from the triplet PES to the singlet PES takes place, whereas the singlet pathways leading to cyclopentadiene + CO, phenoxy + H, and the three-body channel C_5_H_5_ (cyclopentadienyl) + H + CO are highlighted in blue. The schematic triplet/singlet PES depicted in Figure 1, which was first reported (in a somewhat different version) in our recent Letter,^34^ will be used to discuss and rationalize the findings from the present CMB experiments and to understand the detailed mechanism of the O(^3^P, ^1^D) + C_6_H_6_ reactions.

We note that the formation of phenol (channel 3) or cyclopentadiene + CO (channel 2) leads to free-radical chain termination during benzene oxidation at high temperatures; in contrast, the production of phenoxy radical + H (channel 1) provides secondary chain branching. We can therefore expect significant effects of the product BFs on models of benzene oxidation. Notably, there appears to be a significant disagreement between the BFs derived from previous CMB studies^10,34^ and those recently obtained from kinetic investigations with synchrotron radiation.^24^

The paper is organized as follows. In section 2 the experimental and theoretical methods are described. Experimental results with their analysis and theoretical results are presented in sections 3 and 4, respectively. Discussion follows in section 5, while section 6 summarizes the conclusions.

## 2 Methods

### 2.1 Experiment

The title reactions were investigated using the CMB scattering technique with MS detection and TOF analysis.^53−57^ Briefly, two supersonic beams of the reactants were crossed at 90° under single-collision conditions in a large scattering chamber kept at about 2 × 10^–6^ mbar under operating conditions (2 × 10^–7^ mbar base pressure). The angular and velocity distributions of the reaction products were recorded by a triply differentially pumped ultrahigh-vacuum (UHV) (10^–11^ mbar) detector equipped with a tunable electron impact ionizer followed by a quadrupole mass filter and a Daly-type^58^ ion detector. The whole detector unit could be rotated in the plane of the two beams around their intersection axis (Θ = 0° represents the direction of the atomic oxygen beam). The velocities of reactants and products were derived using single-shot and pseudorandom TOF analyses, respectively. Product angular distributions were recorded by modulating the benzene beam at 160 Hz for background subtraction. In the TOF measurements of reaction products, high time resolution was achieved by spinning the pseudorandom TOF disk (provided with four 127-bit pseudorandom sequences), located at the entrance of the detector, at 328.1 Hz (corresponding to a dwell time of 6 μs/channel). The flight length was 24.3 cm.

The supersonic beam of O atoms was produced by a radiofrequency (RF) discharge source^59,60^ operating at an RF power of 300 W on a dilute (5%) gas mixture of O_2_ in He at a carrier pressure of 85 mbar passed through a 0.48 mm diameter water-cooled quartz nozzle followed by a 0.8 mm diameter boron nitride skimmer and a further collimating aperture. In this manner, the atomic oxygen beam mainly contains O(^3^P) and a small amount of O(^1^D) (≤10%).^60^ The peak velocity and speed ratio were 2206 m/s and 4.5, respectively. The supersonic beam of benzene was generated by expanding through a 0.1 mm diameter stainless-steel nozzle, kept at room temperature, neat benzene at 103 mbar maintained in a bath at 290 K to avoid vapor pressure fluctuations. The beam peak velocity and speed ratio were 521 m/s and 4.2, respectively. The resulting collision energy was 8.2 kcal/mol. The small percentage of O(^1^D) present in the atomic oxygen beam was expected to contribute significantly to the measured product distributions because the reaction cross section of O(^3^P) with benzene is considerably lower than that of O(^1^D), as the O(^3^P) + C_6_H_6_ reaction is characterized by a very significant entrance energy barrier of about 4 kcal/mol^34^ while the O(^1^D) reaction is barrierless.^51^

From the laboratory (LAB) angular and TOF distributions at different mass-to-charge (*m*/*z*) ratios, product angular, *T*(θ), and translational energy, *P*(*E*_T_^′^), distributions in the center of mass (CM) system were derived for all channels of the O(^3^P) reaction (channels 1–3) and the O(^1^D) reaction (channels 4–6). For the physical and quantitative interpretation of the scattering data, it is necessary to perform a coordinate transformation and move from the LAB reference frame to the CM frame.^53−56^ For reactions with multiple channels, as in the present work, if more than one product channel contributes to the signal at a given *m*/*z* ratio, a weighted total CM differential cross section reflecting the various possible contributions is used in the data analysis of the LAB angular and TOF distributions for a specific *m*/*z*, that is, *I*_CM_(θ, *E*_T_^′^)_total_ = ∑_*i*_*w*_*i*_[*T*(θ)*P*(*E*_T_^′^)]_*i*_, where the parameters *w*_*i*_, representing the relative contributions of the integral cross sections of the *i*th channel, are best-fit parameters.^18,19,56^ The *T*(θ) and *P*(*E*_T_^′^) functions contain all of the information about the reaction dynamics. The best fit of the LAB product angular, *N*(Θ), and TOF, *N*(Θ, *t*), distributions is achieved by forward convolution of tentative CM distributions over the experimental conditions. Specifically, the CM angular and translational energy distributions are assumed, averaged, and then transformed to the LAB frame for comparison with the experimental distributions, and the procedure is repeated until a satisfactory fit of the experimental distributions is obtained.

### 2.2 Theory

The approach used to investigate the title reaction has been described in our previous studies^34,61^ and therefore is only briefly summarized here. Structures and vibrational frequencies of all stationary points were determined at the unrestricted ωB97X-D/6-311+G(d,p) level. Energies were then calculated at the CCSD(T)/aug-cc-pVTZ level using DF-MP2/aug-cc-pVQZ – DF-MP2/aug-cc-pVTZ-level corrections for basis set size effects. For saddle points with significant multireference character and for barrierless reactions, energies were determined at the CASPT2/aug-cc-pVTZ level. Specifically, the energy barriers of all transition states on the triplet PES and of ^1^W4 decomposition to CO and C_5_H_6_ (^1^TS4) and ^1^TS12. A 0.25 IPEA shift was used in all of the CASPT2 calculations. Details on the adopted active spaces are collected in our previous study.^34^ The structures, frequencies, and energies for all of the calculated stationary points are the same as in our previous study,^34^ except for the H β-scission on the triplet PES, ^3^TS2, whose barrier was re-evaluated using a larger (12e, 11o) active space composed of the oxygen (4e, 3o) p electrons and orbitals, the (6e, 6o) π and π* electrons and orbitals of benzene, and the (2e, 2o) σ and σ* electrons and orbitals of the reacting C–H bond. The barrier so calculated is −0.9 kcal/mol, in good agreement with the value of −1.3 kcal/mol determined at the CCSD(T) level and 1.3 kcal/mol lower than determined in our previous work.^34^

The MECP between the triplet and singlet PESs was determined with energies and analytical gradients evaluated at the ωB97X-D/6-311+G(d,p) level using an unrestricted formalism. The MECP geometry was determined using EStokTP, an open-source software recently developed by us.^62^ The search for the MECP was performed by minimizing the energy on the singlet PES while imposing the constraint that the singlet and triplet energies be equal.^63^ The search for the constrained local minimum was performed via the sequential least-squares quadratic programming (SLSQP) algorithm,^64,65^ which solves the nonlinear minimization problem by producing a sequence of quadratic approximations to the objective function via Broyden–Fletcher–Goldfarb–Shanno (BFGS)-type low-rank updates of the approximate Hessian. At each step, the equality constraints are imposed on the approximate quadratic minimization problem by means of the augmented Lagrangian method.^65,66^

The above method was implemented in EStokTP by interfacing the solver to the open-source optimization library NLopt,^66^ which contains a C language reimplementation of the original Fortran code for SLSQP. Optimizations were started from the ^3^W1 structures and converged within about 50 steps with accuracies of 10^–10^ hartree using tolerances of 10^–4^ hartree. Rate constants of individual channels were determined using conventional transition state theory (TST) and variational TST (VTST). In particular, rate constants for barrierless H loss reactions from the singlet PES were determined using VTST, adopting ωB97X-D/6-311+G(d,p) geometries and frequencies and CASPT2 energies. The rate constant for ISC was determined using nonadiabatic TST (NA-TST). Because in the present work we extend our implementation of NA-TST, which has some specificities compared with other approaches reported in the literature, it is useful to discuss in greater detail how our spin-forbidden microcanonical rate constant was determined. Microcanonical rate constants were computed using our kinetic Monte Carlo stochastic RRKM (MC-RRKM) code at the *E*, *J*-resolved level. For a spin-forbidden reaction in the framework of NA-TST, this means that the rate constant *k*(*E*, *J*) is evaluated as
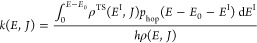
7where ρ^TS^ and ρ are the densities of states of transition state and reactant, respectively, *E*_0_ is the energy of the barrier, *E*^I^ is the energy in the internal degrees of freedom at the MECP, *J* is the angular momentum, *E* is the rovibrational energy, and *p*_hop_ is the probability of intersystem crossing. Energy-resolved rate constants are then computed as suggested by the Miller *E*, *J* model^67^ as
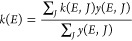
8where *y*(*E*, *J*) is defined as
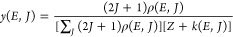
9where *Z* is the intermolecular collision rate. In this model, the energy-dependent rate constant *k*(*E*) is obtained from the *E*, *J*-dependent rate constant *k*(*E*, *J*) by weighting with the *y*(*E*, *J*) distribution obtained under the assumption that the *J* distribution after an intermolecular collision is independent of the angular momentum of the molecule before collision.^67^

The probability of intersystem crossing, *p*_hop_, was determined using two different models. In the double-passage Landau–Zener (LZ) model,^68^*p*_hop_ is calculated as
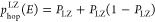
10where the Landau–Zener single-passage ISC probability *P*_LZ_ is given by
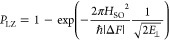
11and in the weak coupling (WC) ISC approximation originally suggested by Nikitin,^69,70^*p*_hop_ is calculated as
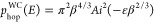
12where
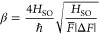
13and
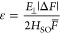
14in which
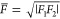
15where *H*_SO_ is the spin–orbit coupling factor, |Δ*F*| is the norm of the difference between the *F*_1_ and *F*_2_ gradients of the two PESs at the MECP calculated in (square root) mass-weighted Cartesian coordinates, *E*_⊥_ is the energy in the hopping coordinate (equal to the *E* – *E*_0_ – *E*^I^ factor), and *Ai* in eq 12 is the Airy function.

The density of states ρ(*E*, *J*) in eq 7 was determined in the rigid rotor–harmonic oscillator (RRHO) approximation. MECP frequencies were determined using the bordered Hessian of the constrained energy function minimized to locate the MECP:
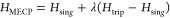
16where *H*_sing_, *H*_trip_ and λ are the Hessians of the singlet and triplet PESs and the Lagrangian multiplier (0 ≤ λ ≤ 1), respectively.

Spin–orbit couplings (*H*_SO_) were evaluated using the state-interacting method at the MECPs using a Breit–Pauli Hamiltonian and a CASSCF wave function.^20^ The T2/S0 *H*_SO_ coupling in this system, square-averaged over the three triplet–singlet coupling elements, is about 35 cm^–1^ for both ISC1 and ISC2, thus exhibiting a small dependence on the MECP geometry.^34^

Master equation simulations were performed with our MC-RRKM code. For the thermal simulations, the intermolecular energy transfer was described using a single-exponential down model,^71^ with the same average collision downward energy transfer (Δ*E*_down_) parameter of 260 × (*T*/300)^0.875^ that we used in the ME investigation of phenol decomposition, assuming that the bath gas is Ar.^61^ The termination threshold for the Monte Carlo simulations was 10^4^ reactive events. All of the DFT calculations were performed using Gaussian 09,^72^ while CCSD(T), DF-MP2, and CASPT2 calculations were performed with Molpro.^73^

## 3 Experimental Results

**Figure 2:**
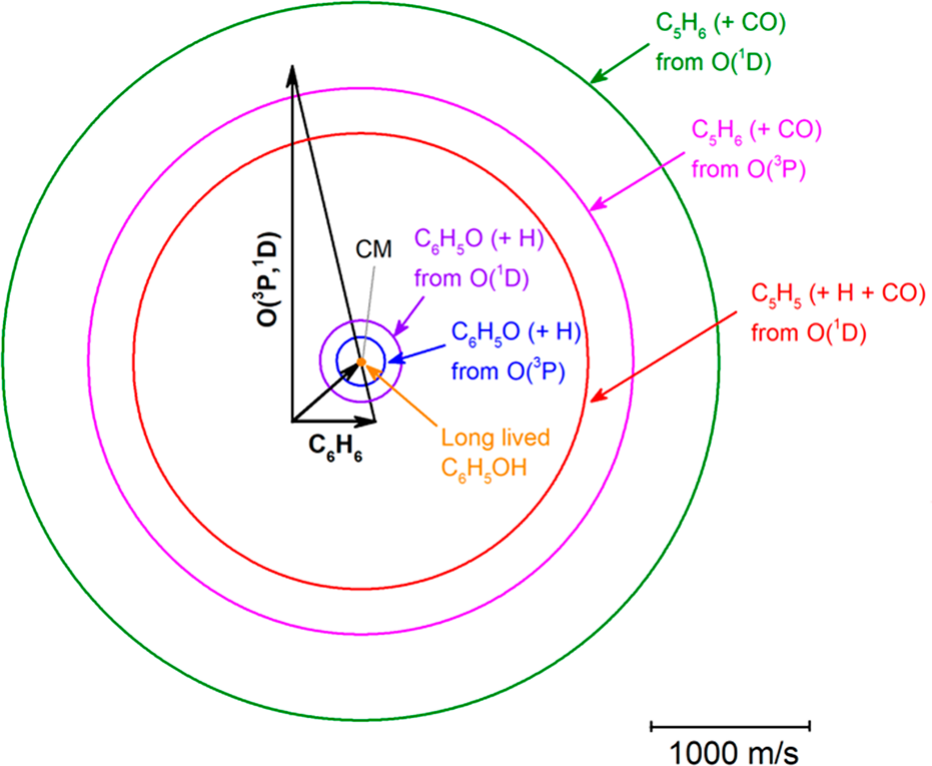
Velocity vector (Newton) diagram of the experiment. The radius of each circle represents the maximum velocity that the indicated product can attain in the CM system if all of the available energy is channeled into product recoil energy. The phenol adduct is centered at the CM (that is, it follows the “centroid’ distribution) and therefore has zero velocity in this frame.

In Figure 2 we depict the velocity vector (so-called Newton) diagram of the experiment on the reactions O(^3^P, ^1^D) + C_6_H_6_. There, the superimposed circles delimit the maximum velocities that the indicated bimolecular primary products can attain with the assumption that all of the available energy (given by *E*_c_ – Δ*H*_0_^0^) is channeled into product translational energy.

Because in this study we observe products from the reactions of benzene with both O(^3^P) and O(^1^D) simultaneously, one important issue is to distinguish their individual contributions. The estimated concentration of O(^1^D) in the beam of atomic oxygen is ≤0.10, that is, it is at least 10 times smaller than that of O(^3^P).^60^ From the PES in Figure 1 we can see that, although O(^3^P) and O(^1^D) can give rise to the same two product channels forming C_6_H_5_O + H and C_5_H_6_ + CO, they will have very different exothermicities and especially very different dynamics. In fact, O(^3^P) can produce phenoxy + H adiabatically on the triplet PES over a significant exit potential barrier but also nonadiabatically on the singlet PES without an exit barrier via ISC from the triplet to the singlet PES. On the other hand, O(^1^D) can produce phenoxy + H adiabatically on the singlet PES with no exit barrier. Moreover, while O(^3^P) can produce C_5_H_6_ + CO only nonadiabatically via ISC, O(^1^D) can produce C_5_H_6_ + CO adiabatically on the singlet PES, and in addition, the C_5_H_6_ product can have enough internal energy to unimolecularly dissociate to C_5_H_5_ + H (three-body process). It should be noted that dissociation of C_6_H_5_O to C_5_H_5_ + CO is energetically not possible for O(^3^P) because of a high barrier of ca. 50 kcal/mol (see Figure 1) and negligible also for O(^1^D).^51^ Furthermore, we note that previous CMB work on the reactions of both O(^3^P) and O(^1^D) with benzene has shown that some phenol adduct survives until the detector for the O(^3^P) reaction^10^ but not for the O(^1^D) reaction.^51^ The phenol adduct observed at the detector can arise from radiative stabilization of the excited phenol intermediate (^1^W1 in Figure 1) or from the fact that phenol has a distribution of lifetimes and a small fraction of it could have a lifetime of ≥300 μs, which would be sufficient to reach the detector. In light of the information from previous studies^10,51^ and taking into account that products arising from the much more exothermic O(^1^D) reactions are expected to fragment more strongly than those from O(^3^P) to daughter ions upon 70 eV electron impact ionization, we were able to exploit the following properties of the scattering to differentiate the contributions of O(^3^P) and O(^1^D) to the total reactive signal: (i) the different reaction energetics, (ii) the different reaction kinematics, (iii) the different reaction dynamics, and (iv) the theoretical information about the triplet and singlet PESs.

**Figure 3:**
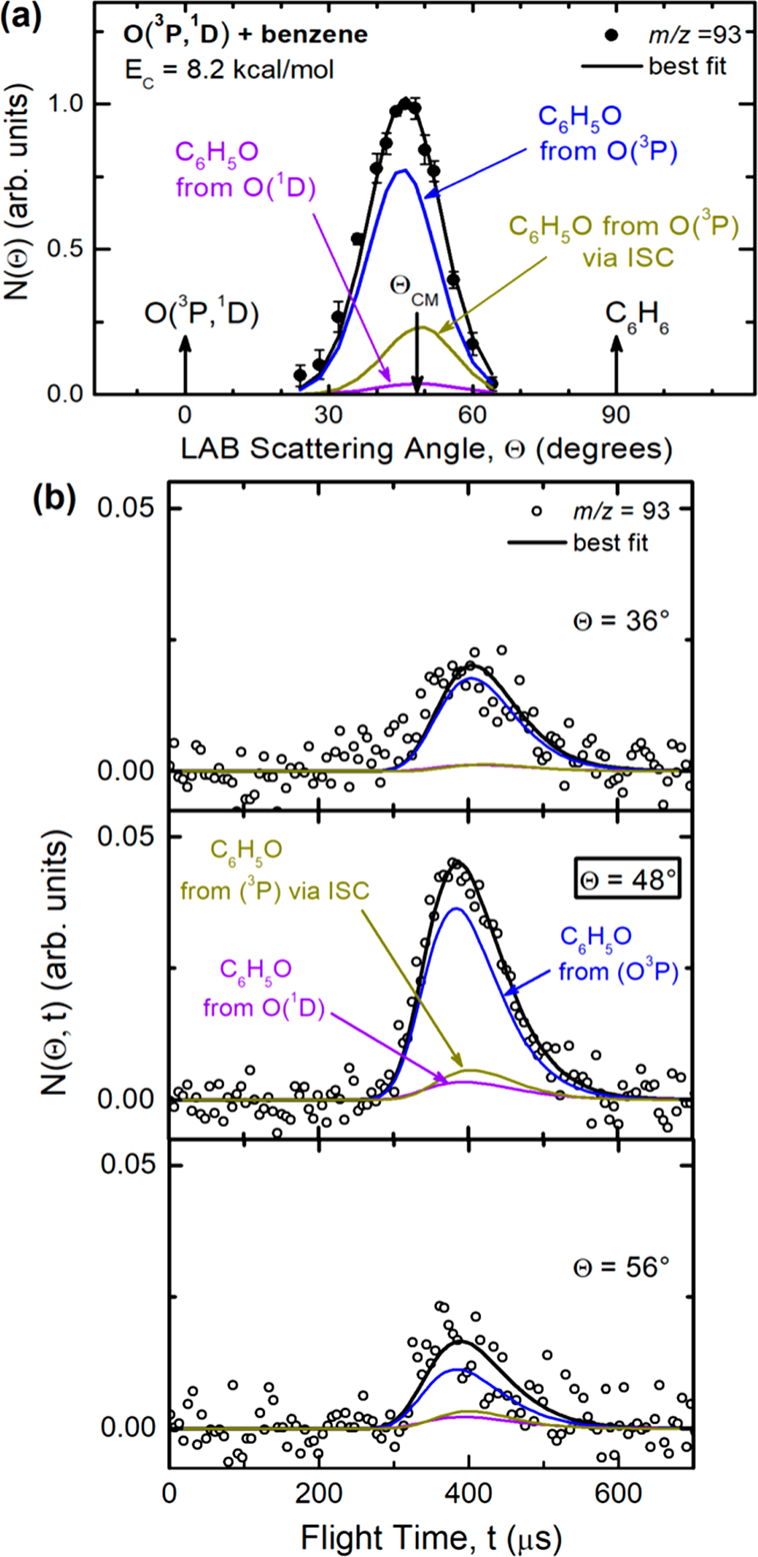
(a) LAB angular distribution and (b) TOF distributions at selected LAB angles for the *m*/*z* = 93 product (phenoxy) from the reactions of O(^3^P) and O(^1^D) with benzene at *E*_c_ = 8.2 kcal/mol. Partial contributions from channels 1 and 4 are represented with color-coded, labeled lines; the black line is the total best fit.

**Figure 4:**
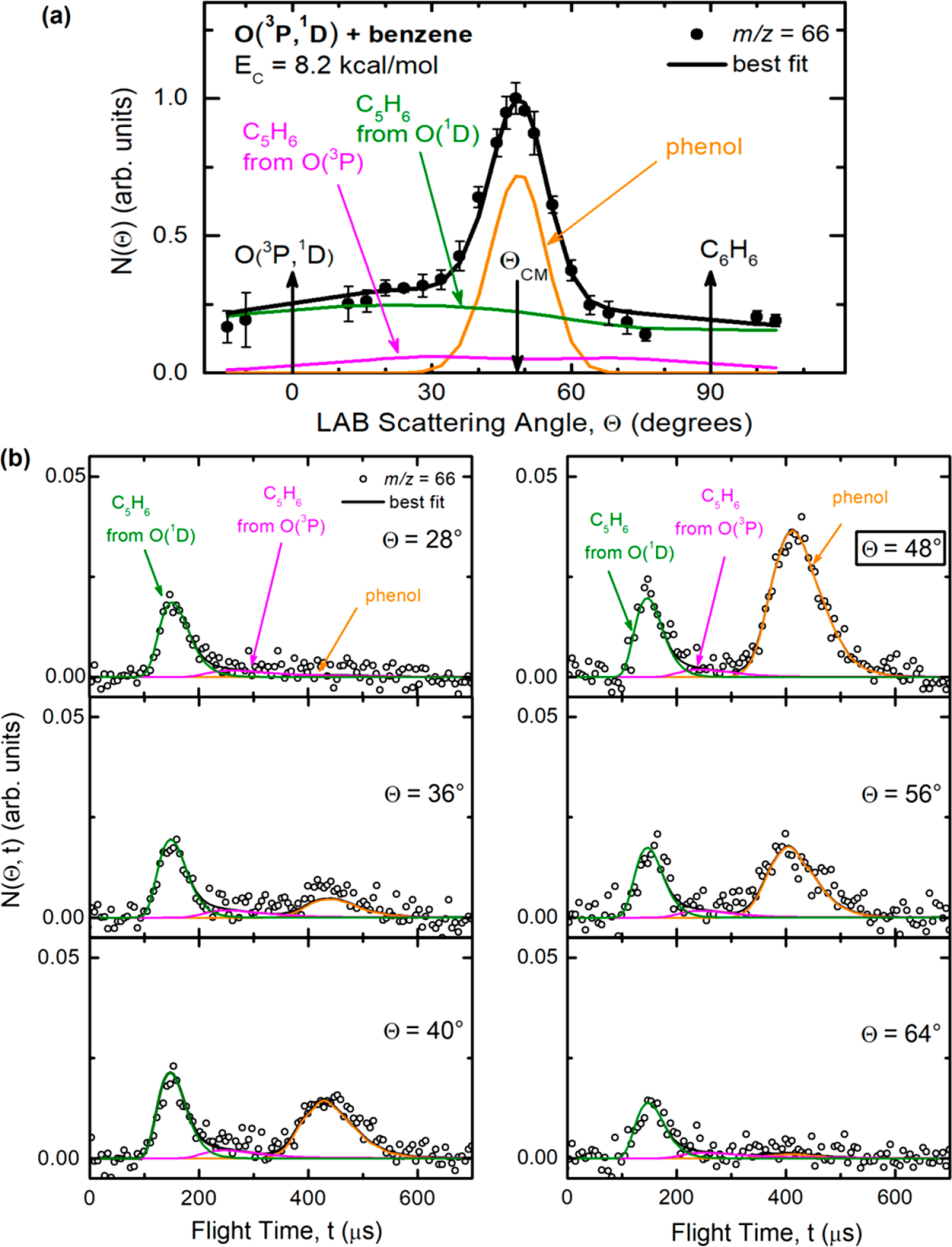
(a) LAB angular distribution and (b) TOF distributions at selected LAB angles for *m*/*z* = 66 products from the reactions of O(^3^P) and O(^1^D) with benzene at *E*_c_ = 8.2 kcal/mol. Partial contributions from channels 2, 3, and 5 are represented with color-coded, labeled lines; the black line is the total best fit.

**Figure 5:**
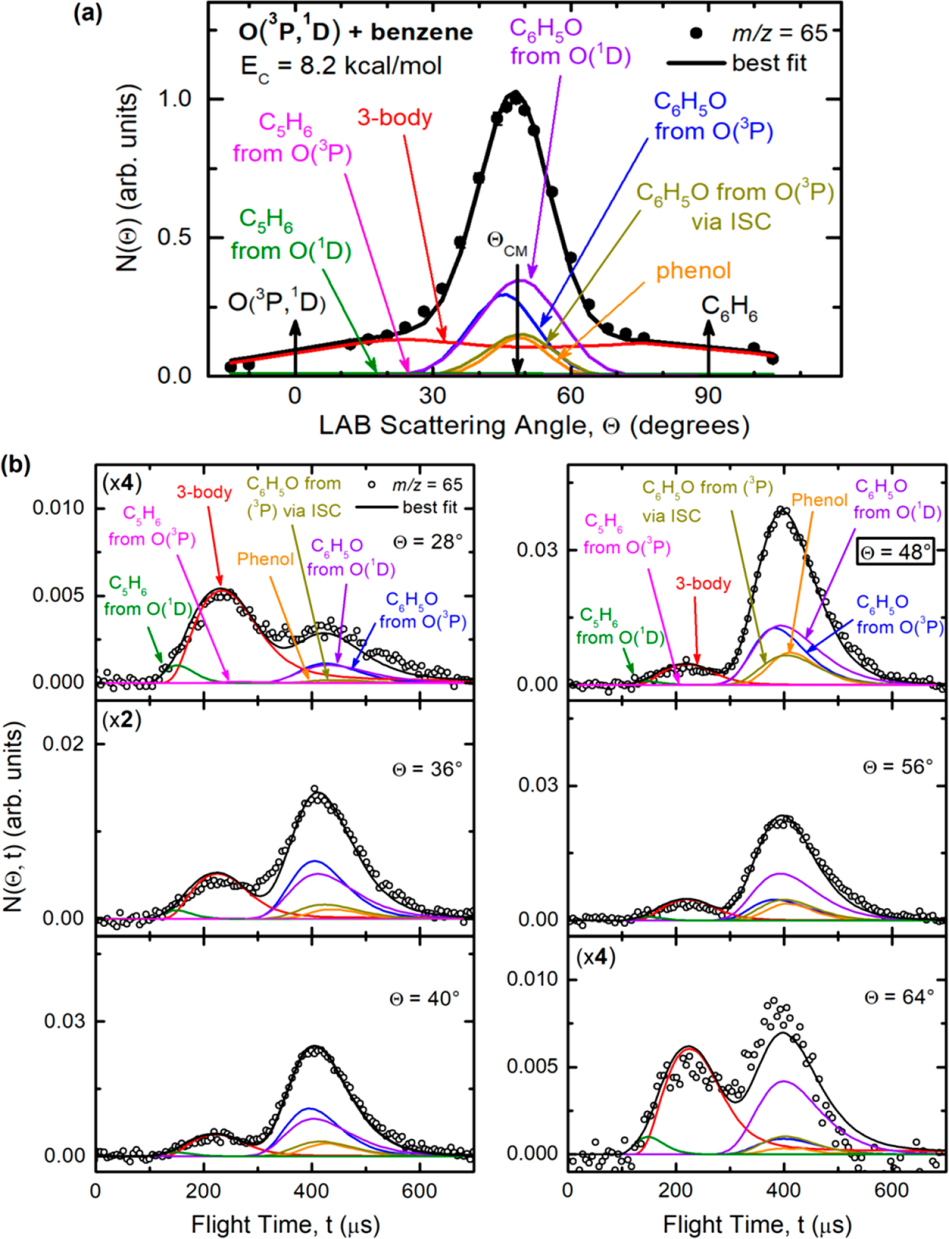
(a) LAB angular distribution and (b) TOF distributions at selected LAB angles for *m*/*z* = 65 products from the reactions of O(^3^P) and O(^1^D) with benzene at *E*_c_ = 8.2 kcal/mol. Partial contributions from channels 1–6 are represented with color-coded, labeled lines; the black line is the total best fit.

Reactive scattering signals were measured at *m*/*z* = 94 (C_6_H_6_O), 93 (C_6_H_5_O), 66 (C_5_H_6_), and 65 (C_5_H_5_) with relative intensities of 0.01, 0.06, 0.08, and 1.00, respectively, when hard ionization detection (70 eV electron energy) was employed (it was neither necessary nor useful to resort to soft ionization detection for this reactive system). The signal at *m*/*z* = 94, when corrected for the ^13^C natural content (6.6%) of the signal at *m*/*z* = 93, corresponds to some phenol adduct that survives from the collision zone until the ionizer of the detector. LAB angular distributions, *N*(Θ), and TOF distributions, *N*(Θ, *t*), were measured for all three masses (*m*/*z* = 93, 66, and 65) corresponding to possible bimolecular products of the O(^3^P,^1^D) + C_6_H_6_ reactions. The angular distributions for *m*/*z* = 93, *m*/*z* = 66, and *m*/*z* = 65 are shown in panels (a) of Figures 3, 4, and 5, respectively, while the TOF distributions at selected LAB angles are presented in the corresponding panels (b) of the same figures.

The various product channels corresponding to H displacement (channels 1 and 4), C_5_H_6_ and C_5_H_5_ formation (channels 2 and 5 and channel 6, respectively), and phenol adduct formation (channel 3) can be disentangled in the TOF measurements, as shown in Figures 4b and 5b. These two figures show the power of TOF analysis in these experiments, whereby different products (arising from different reaction channels) that are detected at the same *m*/*z* value and are usually not clearly distinguishable in the angular distribution at that *m*/*z* (Figures 4a and 5a) are instead separated (or partially separated) according to their different flight times in the TOF distributions at specific LAB angles (Figures 4b and 5b). For each of the three masses at which angular distributions were measured (Figures 3a, 4a, and 5a), the variation of the various channel contributions with the LAB scattering angle Θ is well-exemplified in Figures 3b, 4b, and 5b. For instance, in Figure 4b the strong peak at about 420 μs (corresponding to the CM velocity) for *m*/*z* = 66 is due to the small quantity of phenol (parent mass 94) that survives up to the ionizer and is then dissociatively ionized to C_5_H_6_^+^. Notably, in Figure 4b phenol cannot be observed at Θ = 28° and 64° for kinematic reasons, as these angles fall outside those of the centroid distribution (which is represented by the phenol angular distribution depicted in Figure 4a). While the fast products distributed over much wider Newton circles are due to the cyclopentadiene (C_5_H_6_) product (detected at its parent ion mass) from channels 2 and 5 (from the O(^3^P) and O(^1^D) reactions, respectively), the latter is much faster than the former because of the much larger exoergicity of channel 5 with respect to channel 2. Similarly, in Figure 5a the strong global peak at the CM angle for *m*/*z* = 65 (C_5_H_5_^+^) is due to a small quantity of phenol daughter ion and to the daughter ions of phenoxy from the O(^3^P) and O(^1^D) reactions (channels 1 and 4, respectively), while the fast products distributed over much wider Newton circles are mainly due to the cyclopentadienyl radical (C_5_H_5_) product (detected at its parent mass) from the three-body channel from the O(^1^D) reaction (channel 6) and to a smaller extent to the daughter ion of cyclopentadiene from channels 2 and 5.

**Figure 6:**
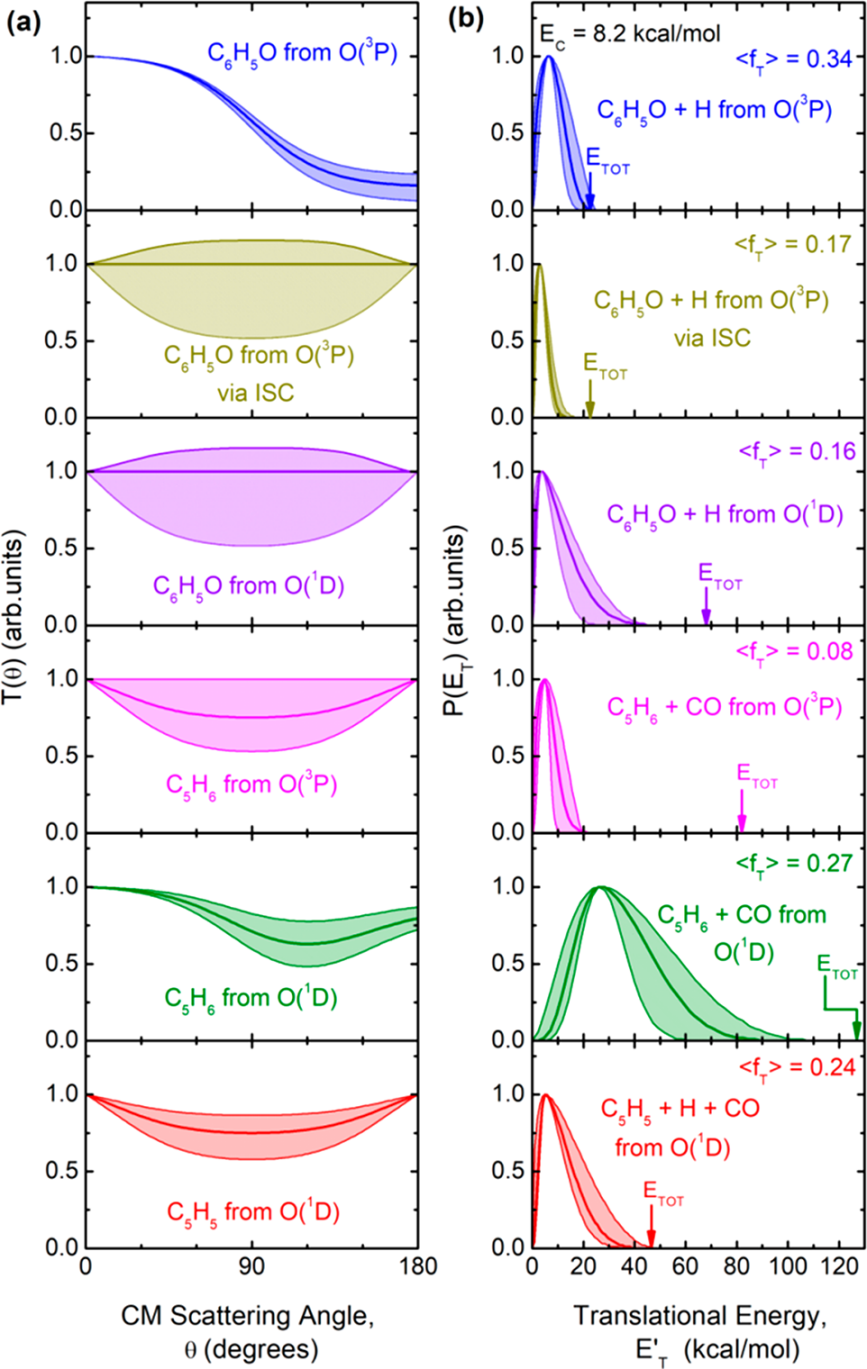
(a) Best-fit CM angular distributions *T*(θ) for the indicated products from the O(^3^P) and O(^1^D) reactions. (b) Corresponding best-fit product translational energy distributions *P*(*E*_T_^′^) for the indicated channels. The total available energy is indicated by the arrow, and the average fraction of energy released in product translation, ⟨*f*_T_⟩, is also given. The shaded areas represent the error bars determined for the CM functions.

The best-fit CM functions *T*(θ) and *P*(*E*_T_^′^) of the LAB angular and TOF distribution data reported in Figures 3–5 for the various product channels are shown in Figure 6. Because the data shown in Figures 3–5 carry the fingerprints of channels 1–3 from O(^3^P) and channels 4–6 from O(^1^D), from the derived best-fit CM functions depicted in Figure 6 we have estimated the corresponding global BFs (see Table 1) and from these also the distinct BFs for the O(^3^P) reaction channels and the O(^1^D) reaction channels (see Table 2). In the following, we analyze the LAB angular and TOF distributions for the various *m*/*z* values to derive the best-fit CM *T*(θ) and *P*(*E*_T_^′^) functions for the various channels.

**Table 1:** Experimental BFs Determined for the O(^3^P, ^1^D) + Benzene Reactions at *E*_c_ = 8.2 kcal/mol^a^

reactants	primary products	reaction channel	PES involved	BF
O(^3^P) + C_6_H_6_	C_6_H_5_O + H	1	triplet	0.023 ± 0.07
	C_6_H_5_O + H	1	singlet via ISC	0.009 ± 0.004
	C_5_H_6_ + CO	2	singlet via ISC	0.015 ± 0.007
	phenol	3	singlet via ISC	0.001 ± 0.0005
O(^1^D) + C_6_H_6_	C_6_H_5_O + H	4	singlet	0.03(5) ± 0.01
	C_5_H_6_ + CO	5	singlet	0.32(4) ± 0.09
	C_5_H_5_ + CO + H	6	singlet	0.59(3) ± 0.15

a The experimental uncertainties vary between 25% and 50% depending on the reactive channel.

**Table 2:** Experimental BFs Determined for the O(^3^P) + C_6_H_6_ and O(^1^D) + C_6_H_6_ Reactions at *E*_c_ = 8.2 kcal/mol, as Obtained from Table 1 (See the Text), Compared to the BFs Predicted by RRKM/ME Calculations on the Coupled Triplet/Singlet PESs at *E*_c_ = 8.2 kcal/mol^a^

			BFs			
reactants	primary products	PES involved	CMB expt^b^	RRKM/ME^b^	lit. expt^c^^,^^d^	RRKM/ME^e^
O(^3^P) + C_6_H_6_	C_6_H_5_O + H	triplet	0.48 ± 0.15	0.26	0.33 ± 0.13^c^	0.46
	C_6_H_5_O + H	singlet via ISC	0.18 ± 0.09	0.15		0.13
	C_5_H_6_ + CO	singlet via ISC	0.32 ± 0.14	0.59	0.33 ± 0.08^c^	0.14
	phenol	singlet via ISC	0.02 ± 0.01	0	0.33 ± 0.08^c^	0.27
O(^1^D) + C_6_H_6_	C_6_H_5_O + H	singlet	0.04 ± 0.02		minor^d^	
	C_5_H_6_ + CO	singlet	0.34 ± 0.10		minor^d^	
	C_5_H_5_ + CO + H	singlet	0.62 ± 0.15		dominant^d^	

a The experimental uncertainties vary between 25% and 50% depending on the reactive channel.

b This work, *E*_c_ = 8.2 kcal/mol.

c For the O(^3^P) reaction, the BFs estimated in experimental kinetic work at 900 K and 4 Torr^24^ are reported and compared with our RRKM/ME predictions.

d For the O(^1^D) reaction, the experimental BFs are compared with those estimated from previous experimental pulsed CMB work at *E*_c_ = 10 kcal/mol.^51^

e This work, 900 K and 4 Torr.

### 3.1 The *m*/*z* = 93 Data: H Displacement Channels

The dynamics of the atomic hydrogen displacement channels leading to phenoxy formation from O(^3^P) (channel 1) and O(^1^D) (channel 4) were characterized by measuring product angular and TOF distributions at different LAB angles for the phenoxy radical parent ion (*m*/*z* = 93) and also for its daughter ion (*m*/*z* = 65). Figure 3a shows that the *m*/*z* = 93 *N*(Θ) is bell-shaped and centered around the CM angle (Θ_CM_ = 48°), but with a clear forward bias, while in Figure 3b the TOF spectra measured at three different LAB angles (the CM angle of 48°, Θ = 36° in the forward direction, and Θ = 56° in the backward direction) exhibit a peak centered at around 380 μs, which is somewhat faster than the peak of the phenol adduct (see Figure 4b). It should be noted that the phenol peak is centered at the CM velocity (since it follows the centroid distribution), while the phenoxy peak, on the basis of energy and momentum conservation, has a velocity somewhat different from the CM velocity because the product translational energy distribution peaks somewhat away from zero (see Figure 6) and the phenoxy loses a very light coproduct (the H atom). Specifically, the TOF peak appears in the LAB frame at velocities larger than the CM velocity, that is, at flight times shorter than that of the CM (i.e., about 420 μs, which is where the phenol peak occurs, as can be seen in the TOF spectrum of phenol at Θ = 48° in Figure 4b).

The primary products at *m*/*z* = 93 were identified as C_6_H_5_O (phenoxy) from the C_6_H_5_O + H channel from both the O(^3^P) and O(^1^D) reactions. Notably, part of the phenoxy yield from O(^3^P) can originate adiabatically from the triplet PES and part non-adiabatically from the singlet PES (via ISC). We have exploited the different energetics, kinematics, and dynamics of the above three H displacement pathways to derive their best-fit CM functions and then estimate their relative contributions. The expected different fragmentation pattern that characterizes hot phenoxy that originated from the O(^3^P) or O(^1^D) reaction was also noted. In fact, phenoxy from O(^3^P) was mainly detected at *m*/*z* = 93 (parent ion), while the presence of phenoxy from the O(^1^D) reaction was mostly observed through its higher fragmentation at *m*/*z* = 65 (Figure 5; see below). Since the heavy product C_6_H_5_O from channel 1 and the corresponding one from the O(^1^D) reaction are kinematically constrained and hence scattered within two small, yet different Newton circles (Figure 2), their intensity is strongly amplified in the LAB frame (Figure 3a) because of the CM → LAB Jacobian transformation.^53−57^ In Figure 3, the distinct contributions from the O(^3^P) and O(^1^D) reactions are represented as color-coded, labeled curves, while the black line, which is in very good agreement with the experimental data, represents the overall best-fit (total C_6_H_5_O product). The corresponding best-fit CM functions for the O(^3^P) and O(^1^D) reactions producing phenoxy + H are shown in Figure 6a,b. The combined fit of the *m*/*z* = 93 and *m*/*z* = 65 data turned out to be sensitive to the different dynamics of phenoxy formation from O(^3^P) when this occurs directly on the triplet PES (for which, given the modest stability and corresponding relatively short lifetime of the initial triplet diradical intermediate ^3^W1(A′) (Figure 1), it is reasonable to expect a short-lived intermediate complex mechanism) or originates via ISC on the singlet PES (where, because of the much larger stability and expectedly long lifetime of the ^1^W7 intermediate, the formation of phenoxy is expected to proceed via a long-lived complex mechanism). Indeed, as can be seen in Figure 6, the *T*(θ) of phenoxy from O(^3^P) + C_6_H_6_ occurring on the triplet PES is strongly forward-biased. In fact, as can be seen in Figure 3a, its partial contribution to the LAB angular distribution peaks at an angle smaller than Θ_CM_ (i.e., in the forward direction with respect to the incoming O atom), while the *T*(θ) for the O(^3^P) reaction proceeding via ISC and that for the O(^1^D) reaction are backward–forward-symmetric with respect to Θ_CM_. The O(^3^P) direct contribution on the triplet PES exhibiting a strongly forward peaked *T*(θ) (see Figure 6a) reflects a strongly osculating complex mechanism, which approaches an almost direct scattering mechanism, while the backward–forward-symmetric *T*(Θ)s of phenoxy from O(^1^D) and from O(^3^P) via ISC reflect a long-lived complex mechanism.^74,75^ The anisotropy of the *T*(θ) of phenoxy from the triplet PES and the symmetry of the *T*(θ) of the other two contributions to phenoxy formation are confirmed by the LAB data at *m*/*z* = 65 and their analysis (see section 3.3).

The best-fit *P*(*E*_T_^′^) of the phenoxy + H channel from the O(^3^P) adiabatic reaction (Figure 6b, top panel) clearly shows that it peaks away from zero translational energy, at about 6.4 kcal/mol, corresponding to an average fraction of total available energy in product translation, ⟨*f*_T_⟩, of 0.34. This indicates the existence in the PES of a significant exit potential energy barrier on the way to products. However, the *P*(*E*_T_^′^) for channel (4) extends to an energy (about 40 kcal/mol) larger than that for channel (1) via ISC (which dies off at about 10 kcal/mol), and this is not surprising given the 45 kcal/mol larger exothermicity of channel (4) with respect to channel (1). Notably, the average fraction of total available energy released in translation, ⟨*f*_T_⟩, is consequently much lower than for the O(^3^P) direct reaction (which has ⟨*f*_T_⟩ = 0.33), being 0.17 for the O(^3^P) reaction via ISC and 0.16 for the O(^1^D) reaction (Figure 6b). We remind the reader that the average product translational energy ⟨*E*_T_^′^⟩ is defined as ⟨*E*_T_^′^⟩ = ∑*P*(*E*_T_^′^)*E*_T_^′^/∑*P*(*E*_T_^′^) and the average fraction of the total available energy (*E*_TOT_ = *E*_c_ – Δ*H*_0_^0^) channeled into translation, ⟨*f*_T_⟩, is defined as ⟨*f*_T_⟩ = ⟨*E*_T_^′^⟩/*E*_TOT_.

### 3.2 The *m*/*z* = 66 Data: Phenol and CO + C_6_H_5_ (Cyclopentadiene) Channels

Let us now move on to examine the angular distribution at *m*/*z* = 66, which is characterized by a prominent peak centered at the CM angle superimposed on two broad wings (Figure 4a). The central peak reflects the phenol adduct from the O(^3^P) reaction that fragments in the ionizer to form C_5_H_6_^+^ (losing a CO molecule), and the measured distribution reflects that of the centroid (that is, it is just determined by the velocity and angular spreads of the two reactant beams and the detector acceptance angle (about 1°)). It should also be noted that phenol was also observed as its parent ion (*m*/*z* = 94). In fact, we observed that the signal at this mass (at the CM angle) was higher than the 6.6% of the signal acquired at *m*/*z* = 93, attributed to the phenoxy-forming channels 1 and 4 and corresponding to the reactive signal of the C_6_H_5_O isotopologue with ^13^C natural abundance. Consequently, after subtraction of the phenoxy ^13^C contributions, the remaining *m*/*z* = 94 signal could only be related to the phenol adduct C_6_H_5_OH having a lifetime longer than its flight time from the collision region to the ionization zone of the detector (i.e., ≥300 μs). It should be noted that formation of phenol is not possible under the single-collision conditions of the present CMB experiments because the total energy is well above that of the possible bimolecular product channels, and therefore, the hot phenol intermediate will ultimately decompose into two product moieties. As we have discussed above, the small fraction of sufficiently long-lived phenol observed is expected to fragment very significantly in the ionizer of the MS detector by loss of a CO molecule, giving a very significant ion signal at *m*/*z* = 66. This can very clearly be seen not only in the angular distribution measured at *m*/*z* = 66 (see Figure 4a), where the peak perfectly centered at the CM angle can only originate from the phenol adduct, but also in the TOF data (Figure 4b), which show that the slow peak in the spectrum (peaking at about 420 μs) has a flight time equal to that of the center of mass. It should be noted that the phenol intensity is strongly amplified in the LAB frame because the C_6_H_5_OH adduct has nominally zero velocity in the CM frame and the mass spectrometer is a number density detector.^10,53−55^

In contrast, the two side wings of the product angular distribution at *m*/*z* = 66 (Figure 4a) unambiguously reflect the formation of C_5_H_6_ + CO from both O(^3^P) (channel 2) (the minor part) and O(^1^D) (channel 5) (the dominant part). As can be seen, the LAB angular distribution of the C_5_H_6_ product is very broad because of linear momentum conservation (cyclopentadiene is left by the heavy CO coproduct). While the O(^3^P) and O(^1^D) contributions to CO formation cannot be distinguished in *N*(Θ), they can readily be disentangled by the TOF measurements because of the much larger exothermicity of channel 5 compared to channel 2. Therefore, C_5_H_6_ formed from O(^1^D) is expected to be much faster than C_5_H_6_ from O(^3^P), as indeed observed experimentally (see the *m*/*z* = 66 TOF spectra in Figure 4b, which show the small O(^3^P) contribution peaking at about 250 μs and the very large O(^1^D) one at about 150 μs, as determined by the very different corresponding *P*(*E*_T_^′^) distributions shown in Figure 6b). The fact that the peak attributed to C_5_H_6_ from O(^1^D) is much more intense than that from O(^3^P) (see Figure 4b), despite the fact that the O(^1^D) concentration in the atomic beam is about an order of magnitude lower than that of O(^3^P), is due to the much larger reactive cross section of the barrierless O(^1^D) reaction with respect to the O(^3^P) reaction. Moreover, while the O(^1^D) reaction forming CO + C_5_H_6_ occurs adiabatically on the singlet PES, the O(^3^P) reaction can lead to C_5_H_6_ + CO only via the non-adiabatic process of ISC, which has a relatively low probability. Notably, although the LAB results obtained here are qualitatively similar to those obtained in the early pioneering CMB study of Sibener et al.^10^ at a comparable *E*_c_, where a small amount of phenol was also observed peaking at the CM, the earlier data^10^ were not analyzed in terms of O(^3^P) and O(^1^D) contributions (although O(^1^D) was known to be present in the atomic oxygen beam) because of lack of sufficient TOF resolution (12 μs/channel TOF spectra and a TOF path of about 17 cm vs 6 μs/channel and 24.3 cm, respectively, in the present study) as well as the more limited LAB angular range in which those earlier data were collected.

The CO-forming channel from O(^3^P) was fitted using a symmetric, slightly polarized CM angular distribution and a *P*(*E*_T_^′^) distribution peaking at about 4.7 kcal/mol and falling to zero at around 20 kcal/mol (see Figure 6b), which witnesses only 8% of the total available energy released into product translation (in turn, this corresponds to a very high internal excitation of the molecular CO and C_5_H_6_ products of about 92% of the total available energy). In contrast, the *P*(*E*_T_^′^) distribution of the C_5_H_6_ + CO channel from O(^1^D) peaks at about 25 kcal/mol and extends up to about 90 kcal/mol, featuring a much larger fraction of the total available energy (*E*_TOT_ ≈ 128 kcal/mol) released in translation (⟨*f*_T_⟩ = 0.28) compared with the corresponding channel from O(^3^P). This clearly indicates that a significant fraction of the internal electronic energy (45.3 kcal/mol) of the atomic reactant is channeled into product translational energy.

It is worth comparing the TOF spectra at *m*/*z* = 66 depicted in Figure 4b with those at the same mass depicted in Figure 1c of ref (51). It should be noted that in Figure 1c of ref (51), features β and γ in the TOF spectrum at *m*/*z* = 66 (reported to be already corrected for the contribution of the O(^3^P) reaction) are due to the naturally abundant ^13^C-isotopic C_5_H_5_^+^ detected with very high intensity at *m*/*z* = 65 and that the correction leaves out only the fast peak α (peaking at about 150 μs) due to C_5_H_6_ from the C_5_H_6_ + CO channel from O(^1^D). In contrast, because our TOF data at *m*/*z* = 66 are already corrected for ^13^C-isotopic C_5_H_5_^+^ but not for the O(^3^P) contribution, O(^3^P) reaction products are also present (phenol and C_5_H_6_, with the former strongly amplified at the CM despite its small contribution with respect to C_5_H_6_ from channel 2). Because we did not have two distinct experiments at the same *E*_c_, one with only a O(^3^P) beam and one with a beam containing both O(^3^P) and O(^1^D) (in equal amounts in the case of Chen et al.^51^), to make a correction for the signal due to O(^3^P) similar to that done by Chen et al. and because our goal is rather to derive the dynamics of the reactions of both O(^3^P) and O(^1^D) with benzene, we have reported data at *m*/*z* = 66 that are already corrected for the ^13^C natural isotopic abundance of *m*/*z* = 65. The peak that we see at about 150 μs in all of our TOF spectra shown in Figure 4b is the analogous to peak α in Figure 1c of ref (51). However, and most notably, between the main fastest and slowest peaks observable in Figure 4b, the TOF spectra indicate a small amount of a further contribution whose velocity is intermediate between that of phenol and that of cyclopentadiene from the O(^1^D) + benzene reaction. Although the intensity of this signal is rather low under the present experimental conditions, at this mass-to-charge ratio it can be unambiguously identified as cyclopentadiene (via its parent ion) derived from the reaction of benzene with O(^3^P) (channel 2).

### 3.3 The *m*/*z* = 65 Data: H Displacement, CO + C_5_H_6_, and CO + C_5_H_5_ + H (Three-Body) Channels

With regard to the experimental data acquired at *m*/*z* = 65, the reactive signal was found to be very large (12.5 times larger than at *m*/*z* = 66, as already mentioned), giving a great deal of information. In particular, the angular distribution was measured in the same angular range where the reactive signal at *m*/*z* = 66 was observed, that is, at LAB angles from −12° to 102°. A first comparison between the angular distributions at *m*/*z* = 66 and 65, reported in Figures 4a and 5a, respectively, indicates that the oxidation of benzene occurs through different mechanisms with O(^3^P) and O(^1^D) reactants, which can be discerned from TOF measurements and by analysis of the different fragmentation patterns of the primary products detected at these *m*/*z* ratios. For instance, in both cases the angular distributions are characterized by a central peak. However, in the distribution acquired at *m*/*z* = 65, the peak is wider than that observed at *m*/*z* = 66, especially in the forward direction. It should be noted that the phenoxy product from channels 1 and 4 easily loses a CO molecule in the ionizer to give the *m*/*z* = 65 daughter ion. Moreover, in the angular distributions recorded at *m*/*z* = 66 and 65, there are two wings that extend over a wide LAB angle range. However, at *m*/*z* = 66 a somewhat higher intensity was found in the forward direction, while at *m*/*z* = 65 the relative intensities of the two wings are comparable. This indicates that different primary products contribute at the two mass-to-charge ratios. In particular, the main difference between the two angular distributions (Figures 4a and 5a) is due to the three-body channel from O(^1^D) + benzene (channel 6), producing CO + H + C_5_H_5_, whose occurrence was assessed by the detection of the C_5_H_5_ species via its parent ion at *m*/*z* = 65.

As discussed by Chen et al.^51^ in the analogous pulsed CMB experiments at *E*_c_ = 10 kcal/mol, we note that for the three-body channel (channel 6), the momentum exerted by the H atom is negligible because of its small mass; we can then analyze only the momentum-matching condition for C_5_H_5_ and CO. Because there is no reverse barrier for the H atom loss process from C_5_H_6_ (see Figure 1), the kinetic energy of the H atom product is expected to be small. Since the momentum of the H atom would be small with respect to that of C_5_H_5_ and CO products, in the data analysis we only used the masses of C_5_H_5_ and CO products, neglecting the translational energy of the H atom, as previously done by Chen et al.^51^ We remark that the *T*(θ) distribution for the three-body channel is backward–forward-symmetric and slightly polarized (see Figure 6a), which indicates a reaction mechanism associated with a long-lived complex.^74,75^

Besides the three-body channel from O(^1^D), phenol (channel 3), phenoxy (channels 1 and 4), and cyclopentadiene (channels 2 and 5) also contributed at *m*/*z* = 65 via their daughter ions. Cyclopentadiene is assumed to give an (*m*/*z* = 65)/(*m*/*z* = 66) intensity ratio of about 0.5.^76^ Finally, if we compare the TOF spectra acquired at *m*/*z* = 66 and 65 with those recorded at *m*/*z* = 93, we note that the relative intensity of the peaks changes with varying LAB angle of detection, especially focusing on the slower peak, whose maximum intensity occurs in proximity of Θ_CM_ = 48°. This trend can be explained by considering that products originating from the breakage of the C–C bond of the aromatic ring are more exothermic and, therefore, by linear momentum conservation, scatter over wider Newton circles compared with species derived from H displacement channels, which are kinematically constrained within small circles and therefore have an intensity that is strongly amplified at the CM angle (see Figure 2). In particular, in Figure 5b the large, slow peak centered at around 400 μs originates from phenol (channel 3), and phenoxy from O(^3^P) (channel 1) and O(^1^D) (channel 4). The faster and rather strong peak at around 230 μs is mainly due to C_5_H_5_ from the three-body channel (channel 6), in agreement with the results of ref (51). The fastest shoulder (particularly well visible at Θ = 28°; also see Figure S1) is due to C_5_H_6_ from the O(^1^D) reaction (channel 5), in agreement with Chen et al.^51^ Because of the dominant contribution of the three-body channel, the C_5_H_6_ product contribution from channel 2 is very weak in these *m*/*z* = 65 TOF spectra. Overall, we note the excellent agreement with the results of Chen et al.^51^ on the O(^1^D) + C_6_H_6_ reaction.

We now wish to show that the simultaneous best-fit of the *m*/*z* = 93 and 65 data, with the constraint of reproducing accurately the width and position of the main peaks in the angular distributions around the CM and the shape and relative intensity at the various LAB angles of the corresponding overall peaks in the TOF distributions, has permitted us to derive unambiguously the best-fit CM functions for three contributing pathways to phenoxy formation. The LAB angular distributions at *m*/*z* = 93 (Figure 3a) and 65 (Figure 5a) clearly show that the contributions to the total angular distributions of phenoxy from O(^3^P) via ISC and from O(^1^D) both peak near the CM, while the contribution of phenoxy from O(^3^P) via the triplet PES exhibits a peak at angles smaller than Θ_CM_ (i.e., in the forward direction). Notably, the relative weights of the O(^1^D) and O(^3^P) contributions are opposite at the two masses, with that of O(^1^D) at *m*/*z* = 65 being comparatively much larger than that at *m*/*z* = 93 because hotter phenoxy from O(^1^D) expectedly fragments more extensively in the ionizer. It is useful to examine also the relative contributions in the TOF spectra at *m*/*z* = 65 (where the signal is strongest and the signal-to-noise ratio highest). An examination of the TOF spectra at *m*/*z* = 65 in the forward direction (Θ = 28°), at the CM (Θ = 48°), and in the backward direction (Θ = 64°) shows that the peak of phenoxy from O(^3^P) via ISC peaks closer to the CM velocity (the phenol velocity; see the *m*/*z* = 66 TOF) than the phenoxy from O(^3^P) occurring adiabatically on the triplet PES or from O(^1^D) and that it goes nearly to zero at Θ = 28° because very little energy goes into translation for this channel. At the same time, because of the strong forward peaking of *T*(θ) for phenoxy from O(^3^P) on the triplet PES and the fact that a large fraction of the total available energy goes into translation for this channel (⟨*f*_T_⟩ = 0.34), its contribution appears at Θ_CM_ = 48° and in the forward direction (Θ = 28°), while it is very weak in the backward direction (see the TOF at Θ = 64 in Figure 5b).

### 3.4 Branching Fractions

After the characterization of the CM *T*(θ) and *P*(*E*_T_^′^) functions for the various product channels (Figure 6), the branching fraction of each primary product was estimated using the procedure introduced by Schmoltner et al.^11^ and widely employed by us in the study of a variety of multichannel reactions of O(^3^P) with UHs.^19,22^ The experimental BFs for the competing product channels of the O(^3^P, ^1^D) + benzene reactions at *E*_c_ = 8.2 kcal/mol are listed in Table 1, and the BFs for the distinct O(^3^P) and O(^1^D) reactions are reported in Table 2. The distinct BFs in Table 2 were obtained from Table 1 by simply normalizing to unity, separately, the sum of BFs of all O(^3^P) channels and of all O(^1^D) channels. In Table 2 the BFs for the O(^3^P) reaction are compared with the theoretical predictions from RRKM/ME simulations on the coupled triplet/singlet PES for the conditions of the CMB experiment. In addition, the BFs derived from kinetic studies^24^ at 900 K and 4 Torr are reported, compared with the RRKM/ME predictions for the same conditions from the present study.

It is useful to take a closer look at the BFs in Table 1. If we add all of the yields from the O(^3^P) reaction channels (channels 1–3) and those from the O(^1^D) reaction channels (channels 4–6), we find the following ratio: [yield O(^3^P) reactions]/[yield O(^1^D) reactions] = 0.048/0.952, that is, under our experimental conditions only about 5% of the total reactive signal originates from the O(^3^P) reaction with benzene, while the rest comes from the O(^1^D) reaction. If we assume that the concentration of O(^1^D) in the atomic oxygen beam is about 10% (upper limit) of that of O(^3^P),^60^ this would indicate that at *E*_c_ = 8.2 kcal/mol the total reactive cross section of the reaction of benzene with O(^1^D) is about 190 times larger than that with O(^3^P). This is plausible given that the O(^3^P) + benzene reaction has *k*_300 K_ ≈ 1 × 10^–14^ cm^3^ molecule^–1^ s^–1^ (*k*_900 K_ ≈ 3 × 10^–12^ cm^3^ molecule^–1^ s^–1^)^24^ while the barrierless O(^1^D) reaction with benzene is expected to be gas-kinetic (*k*_300 K_ ≈ 1 × 10^–10^ cm^3^ molecule^–1^ s^–1^, with *k* decreasing only slightly with increasing temperature). Despite the relatively small fraction of the total reactive signal coming from the O(^3^P) reaction, we were able to derive the detailed dynamics also for the O(^3^P) + benzene reaction along with that of the O(^1^D) reaction. The dynamics for the two reactions will be compared with previous findings in the Discussion.

As shown in Table 2, the trends of the BFs for the various channels of the O(^3^P) + benzene and O(^1^D) + benzene reactions are found to be significantly different. For example, for O(^3^P) + benzene the, H displacement channel (channel 1) is dominant (overall BF = 0.66 ± 0.24), while the analogous channel for O(^1^D) + benzene (channel 4) is minor (BF = 0.04 ± 0.02). On the other hand, if we compare channels 2 and 5, we find that C_5_H_6_ + CO formation is significant for both O(^1^D) + benzene (BF = 0.34 ± 0.10)) and O(^3^P) + benzene (BF = 0.32 ± 0.14). Notably, we detected the adduct from the O(^3^P) + benzene reaction (channel 3) as a minor product (BF = 0.02 ± 0.01), and we confirmed that the three-body channel (channel 6) is dominant in the O(^1^D) + benzene reaction (BF = 0.62 ± 0.15), the latter corroborating the results by Chen et al.^51^ However, in contrast to Chen et al., for the O(^1^D) + benzene reaction, we find a reversed yield of the channels forming C_6_H_5_O + H and C_5_H_6_ + CO, despite the fact that the LAB data appear to be very similar. In particular, the branching ratio of [CO + stable C_5_H_6_]/[CO from the three-body channel] is 0.55 ± 0.20 in our case, while it was reported to be 0.12 ± 0.03 in the previous study.^51^ In contrast, the branching ratio of channels [C_6_H_5_O + H]/[three-body channel] is 0.065 ± 0.025 in our case, while it was 0.38 ± 0.06 in the previous study. We do not know the origin of this discrepancy, which is not expected to be justified by the 2 kcal/mol difference in the collision energies of the two experiments. However, it should be noted that even if we add the H channel yield from O(^3^P) to the H yield from O(^1^D), the above large disagreement between the branching ratios from our study and those from the Chen et al. study would persist because the yields of phenoxy + H from O(^3^P) and O(^1^D) are comparable in our study (see Table 1). Interestingly, we note that a reasonable agreement between the two studies, considering the somewhat different *E*_c_, would be obtained if the branching ratios in Chen et al.’s study were actually interchanged, i.e., 0.12 ± 0.03 for the [H]/[three-body] channels (vs our 0.065 ± 0.025) and 0.38 ± 0.06 for the [C_6_H_5_ + CO]/[three-body] channels (vs our 0.55 ± 0.20).

## 4 Theoretical Results

### 4.1 Branching Fractions under CMB Conditions

**Figure 7:**
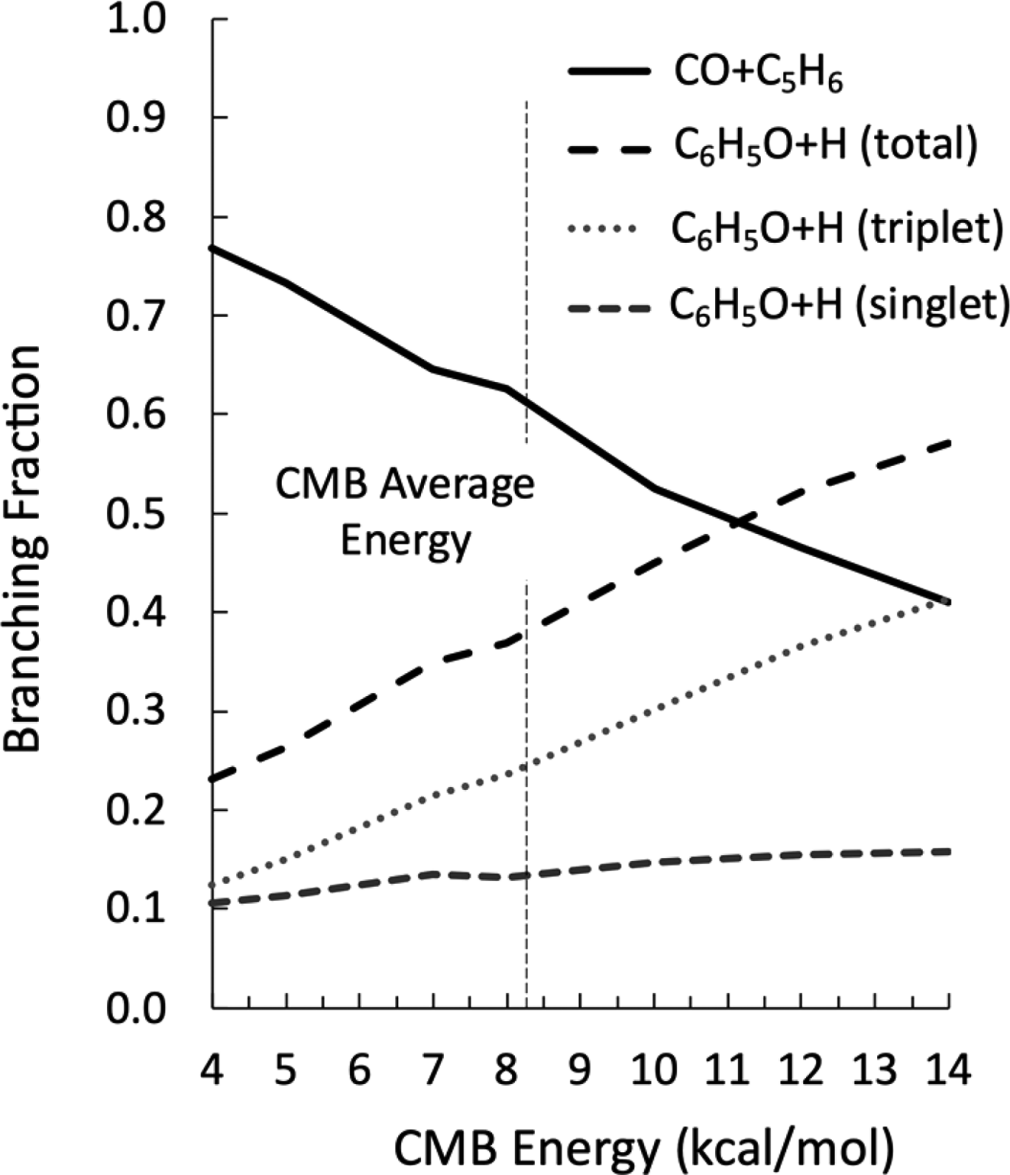
Branching fractions for the reaction between O(^3^P) and benzene computed at different CMB collision energies. The vertical line corresponds to the nominal collision energy (*E*_c_) of the present experiments (8.2 kcal/mol).

The theoretical methodology described in section 2.2 was used to calculate the BFs for the reaction between O(^3^P) and benzene. The calculations were performed using the same procedure as adopted in our previous theoretical studies of reactions between O(^3^P) and unsaturated hydrocarbons (see, e.g., ref (22)), which in general yielded good agreement with experiments. In this instance, however, stochastic kinetic Monte Carlo simulations were performed to account explicitly for the collisional energy distribution in CMB experiments using experimental data to weight collision energy contributions within a 4–14 kcal/mol range with an average value of 8.2 kcal/mol. The weak coupling model was used to compute crossing probabilities at the MECP, and NA-TST theory was employed to evaluate rate constants. The computational results are compared with experimental values in Table 2. It should be noted that the theoretical results differ slightly, by up to a factor of 1.2 in the BFs, from those reported in our previous study^34^ because of the change of the energy barrier of ^3^TS2, the use of the weak coupling ISC model, and the simulation of CMB experiments using the collisional energy distribution rather than the average value. The results of simulations performed at values of the collision energy of the CMB experiment between 4 and 14 kcal/mol, and thus in the range corresponding to the experimental spread of the relative collision energy, are shown in Figure 7. It can be noted that the BFs are significantly sensitive to the relative energy of the beams. When it is low, the main products are C_5_H_6_ and CO. As the collision energy increases, the relevance of this channel progressively decreases because of competition from the H loss channels from the triplet and singlet PESs. It is interesting to notice how H loss from the singlet PES is significant under all conditions, although H loss from the triplet PES is largest even at the lowest CMB energies explored here. As can be observed, there is some disagreement between the experimental and calculated BFs (see Table 2). Experimentally, it is found that the main reaction channel leads to the formation of C_6_H_5_O + H, while theoretically the opposite is true (CO + C_5_H_6_ is larger than C_6_H_5_O + H). However, the relative contributions to the H channel from the triplet and singlet PESs are nicely captured by the model, which predicts that H is produced mainly from the triplet PES. In addition, it should be noted that there is considerable uncertainty in both the experimental determinations and the theoretical calculations. In particular, we observe that the theoretical calculations rely on the ergodic assumption that the relative kinetic energy of the beams following O(^3^P) addition is distributed among all of the molecular degrees of freedom. Our previous investigations of O(^3^P) reactions with unsaturated hydrocarbons suggest that this may not always be the case for H loss channels. To determine the level of uncertainty in the present theoretical calculations, we performed additional calculations using a higher level of theory to compute ISC crossing rates (section 4.2) and checked the impact of selected model parameters on the BFs (section 4.3).

### 4.2 Landau–Zener and Weak Coupling ISC Models

**Figure 8:**
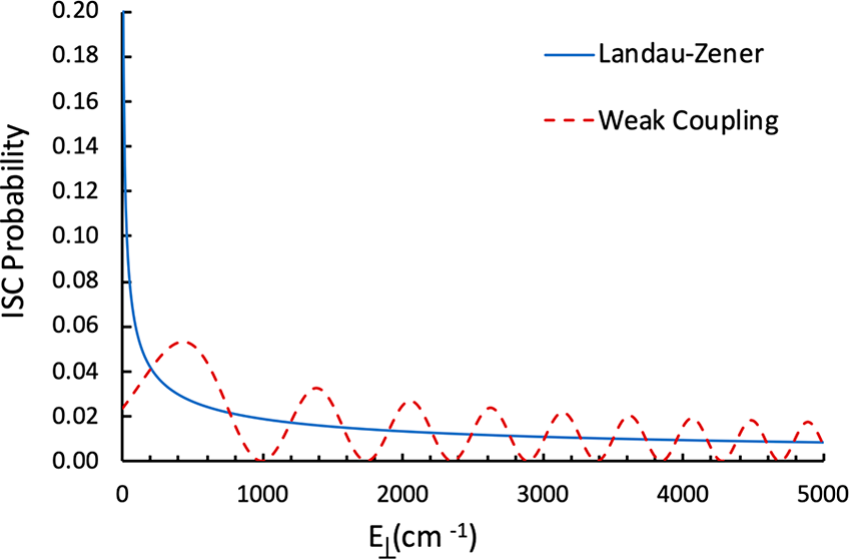
ISC probabilities computed as functions of the energy in the reaction coordinate *E*_⊥_.

**Figure 9:**
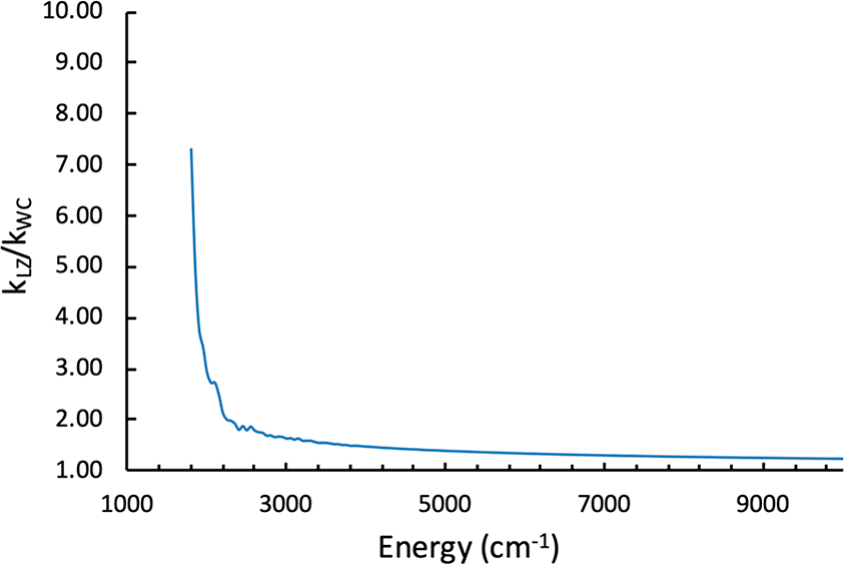
Ratio of ISC rate constants calculated using the LZ and WC models as a function of the total internal energy.

Intersystem crossing probabilities and rate constants were computed at two levels of theory: the Landau–Zener (LZ) model, which is often used to study spin-forbidden processes, and the weak-coupling (WC) model (described in Methods). The WC model is expected to give a better theoretical description of ISC than the LZ model, which tends unphysically to a crossing probability of 1 when the energy in the reaction coordinate (*E*_⊥_) goes to zero and *H*_SO_ is small, as is the case in the present system. A comparison of the ISC probabilities calculated with the two models as a function of *E*_⊥_ is shown in Figure 8. It can be observed that the LZ model, as expected, considerably overestimates the crossing probability at low *E*_⊥_. The two ISC probabilities first cross at *E*_⊥_ = 200 cm^–1^ (≈0.6 kcal/mol). The ratio of rate constants calculated with eq 8 using the LZ and WC models is plotted in Figure 9 as a function of the total internal energy, referenced to the bottom of the reactant well ^3^W1 (see Figure 1).

As could be expected, it can be noted that the LZ rate constant is considerably larger than the WC rate constant at low energies and that the rates become comparable as the energy increases. The impact of using ISC rate constants calculated using the LZ and WC models on the system reactivity is discussed in the next section.

### 4.3 Impact of Model Parameters on Predicted Branching Fractions

The impact of uncertainties and theoretical model approximations on the BFs predicted through master equation simulations is investigated here. In particular, we focus on the theoretical model used to determine the ISC rates (LZ vs WC), on the spread of the collision energy in the CMB experiment, and on uncertainties in the energy of some key stationary points of the PES. The results of this analysis are summarized in Table 3. It can be observed that performing the simulations using the LZ model instead of the WC model has only a small impact on the predicted BFs. The reason for this is that following O(^3^P) addition to the aromatic ring, with the assumption that the collision energy is distributed statistically among the internal degrees of freedom of the intermediate ^3^W1, the average internal molecular energy is about 8000 cm^–1^, which is in the region where the LZ and WC ISC rates are similar (see Figure 9). Simulations were performed using the average collision energy of 8.2 kcal/mol. The data reported in Table 3 can also be used to evaluate the impact of performing the simulations using the CMB collisional energy distribution or the average energy. As can be observed, the impact is small but not negligible.

**Table 3:** Sensitivity of the Calculated Branching Fractions for the O(^3^P) + Benzene Reaction to the CMB Conditions, the Adopted Theoretical Model, and Uncertainties in the Model Parameters

model	C_6_H_5_O + H (triplet)	C_5_H_6_ + CO	C_6_H_5_O + H (singlet)
collision energy distribution	0.26	0.59	0.15
Landau–Zener ISC^a^	0.22	0.64	0.14
weak coupling ISC^a^	0.25	0.61	0.14
MECP energy – 2 kcal/mol^a^^,^^b^	0.10	0.73	0.18
MECP energy + 2 kcal/mol^a^^,^^b^	0.53	0.38	0.08

a Simulations were performed at the average collision energy of 8.2 kcal/mol.

b Simulations were performed using the weak coupling ISC model.

We then investigated the effect of uncertainty in key energy barriers on the predicted BFs. Specifically, we focused on the energy barrier for the reaction of H loss on the triplet PES (^3^TS2) and on the MECP energy. Considering the multireference character of the MECP and the level of the theoretical calculations, it is reasonable to expect that the energies of both of these stationary points may have an uncertainty of at least 1 kcal/mol. As the two reaction pathways are in competition, the impact of these uncertainties was investigated by modifying the energy of the MECP by ±2 kcal/mol, thus condensing the whole uncertainty in this parameter. The results of the simulations indicate a significant effect on the BFs, with the triplet H loss channel BF changing by a factor of 2 and the C_5_H_6_ + CO and singlet H loss channels being modified by about ±0.15. It is therefore reasonable to expect significant sensitivity of the model to the computed energy barrier for the triplet H loss and to the MECP energy and smaller sensitivity for the singlet channels. It should be noted that the BF of the triplet channel is the one where the difference between calculated and experimental data is highest (see Table 2). We note that the agreement between the experimental BFs and the theoretical predictions would improve significantly if the MECP energy were to be increased by 2 kcal/mol, from which the BF(C_6_H_5_O + H from singlet) would become 0.08 (vs 0.18 ± 0.09 experimental), the BF(C_5_H_6_ + CO) would become 0.38 (vs 0.32 ± 0.14), and the BF(C_6_H_5_O + H from triplet) would become 0.53 (vs 0.48 ± 0.15).

### 4.4 Thermal Rate Constants: Pressure Dependence and Fits

**Figure 10:**
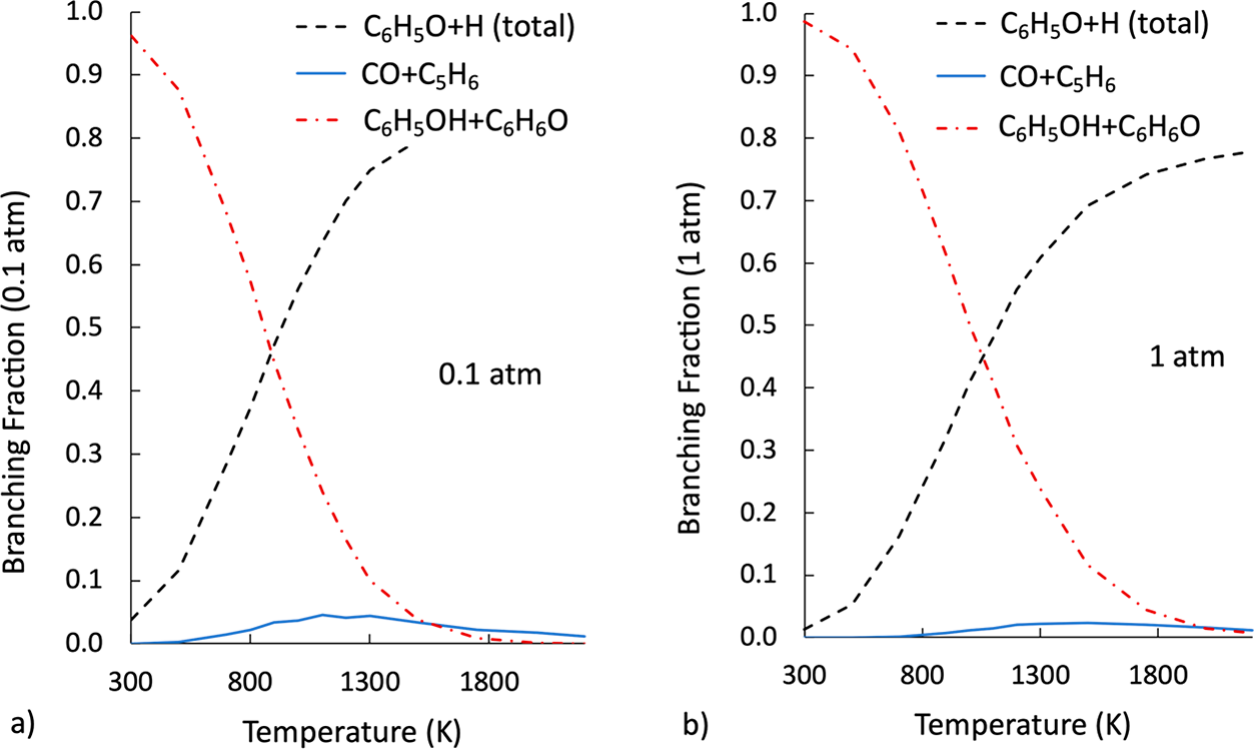
Branching fractions for the reaction between O(^3^P) and benzene computed as functions of temperature at different pressures.

Rate constants were computed as a function of temperature at different pressures using the approach described in Methods and in our previous study.^34^ Because of the inherent relevance in combustion systems, it is interesting at this point to discuss the dependence of the BFs of the main reaction channels on temperature and pressure in greater detail. The BFs calculated at 0.1 and 1 atm are reported in Figure 10. The temperature dependence of the BFs predicted for the thermal simulations in Figure 10 can be compared with that for the CMB simulations performed as a function of the CMB energy reported in Figure 7. It can be noted that the relevance of the BF of the CO + C_5_H_6_ channel is much smaller in the thermal simulations than in the CMB simulations. This is determined by the impact of collisional stabilization of the singlet wells, most notably phenol (^1^W1) and two cyclohexadienone isomers (^1^W2 and ^1^W6), which become the main products on the singlet PES (see Figure 1). Arrhenius fits of rate constants computed at different temperatures and pressures are reported in Table 4.

**Table 4:** Arrhenius Fits in the Form *AT*^α^ exp(−*E*_A_/*RT*)^a^

*P* (atm)	*A* (cm^3^ mol^–1^ s^–1^)	α	*E*_A_ (cal/mol)	*R*^2^	*T* range (K)
C_6_H_6_ + O → C_6_H_5_O + H					
0.1	1.34 × 10^8^	1.76	5620	1.00	300–2200
1	1.04 × 10^8^	1.79	5600	1.00	300–2200
10	2.91 × 10^8^	1.66	5880	1.00	300–2200
100	6.67 × 10^9^	1.28	6930	1.00	300–2200
C_6_H_6_ + O → CO + C_5_H_6_					
0.1	6.09 × 10^15^	–0.91	9840	0.99	300–2200
1	2.22 × 10^18^	–1.58	14800	1.00	300–2200
10	3.01 × 10^19^	–1.83	20300	1.00	800–2200
100	5.63 × 10^02^	2.87	12900	0.99	1000–2200
C_6_H_6_ + O → C_6_H_5_OH (Original)					
0.1	1.80 × 10^16^	–6.73	–14031	0.98	300–1500
1	3.49 × 10^12^	–0.78	1200	0.99	300–1500
10	1.97 × 10^23^	–3.11	8560	1.00	500–1750
100	5.61 × 10^21^	–2.63	7900	1.00	300–2000
C_6_H_6_ + O → C_6_H_5_OH (Duplicate)					
0.1	1.40 × 10^38^	–7.68	14400		
1	1.05 × 10^28^	–4.55	10500		
10	1.46 × 10^32^	–6.88	7500		
100	7.90 × 10^33^	–7.60	7500		

a Units: cal, mol, s, cm^3^. In the fitting, the rate constants for all of the collisionally-stabilized wells (mainly ^1^W1, ^1^W2, and ^1^W7) were merged into that of phenol (^1^W1).

## 5 Discussion

The experimental results will be discussed in light of the calculated triplet and singlet PESs and related theoretical predictions of product BFs from RRKM/ME calculations with inclusion of ISC. In particular, the shape of the CM product angular distributions and the fraction of the total available energy released as product translational motion for each reactive channel will be discussed with analysis of the characteristics of the PESs involved (see Figure 1 and ref (34)) in order to achieve a comprehensive picture of the reaction mechanism.

### 5.1 Product Angular Distributions and Lifetimes of Intermediates

As may be seen in Figure 6, the best-fit CM *T*(θ)s are backward–forward-symmetric for all of the observed products of the O(^3^P) and O(^1^D) reactions except for the phenoxy product from the direct O(^3^P) reaction occurring on the triplet PES. It should be noted that all of other product channels occur on the singlet PES (see Figure 1), and their backward–forward-symmetric *T*(θ)s indicate that the reactions proceed via a long-lived complex mechanism,^74,75^ with intermediate complexes that live at least five or six rotational periods (which are on the order of picoseconds) before decomposing to products. Indeed, the RRKM/ME-computed lifetimes of ^1^W7 and ^1^W2 singlet intermediates (see Figure 1) are on the order of more than 100 ps, which corroborates the long-lived complex mechanism. In contrast, the lifetime of the ^3^W1(A′) intermediate, which can lead adiabatically to the phenoxy + H products, having a rather shallow well of −12.8 kcal/mol, is much shorter (about 10 ps at *E*_c_ = 8.2 kcal/mol and 7 ps at 800 K) than the lifetimes of the singlet intermediates. Consequently, for a range of impact parameters, a large fraction of the reactive O(^3^P) + benzene collisions proceed nearly directly, that is, via a strongly osculating complex mechanism,^74,75^ as witnessed by the strongly forward-biased angular distribution of phenoxy (Figure 6a, top panel). As observed previously in reactions of O(^3^P) with UHs, in numerous cases the H displacement channel was found to behave nonstatistically, that is, if the energy following O attack on the carbons of the aromatic ring is not fully randomized within the triplet complex, the triplet dynamics may not be treatable by statistical theories. Indeed, the BF of the adiabatic H displacement channel from O(^3^P) + benzene is somewhat underestimated by the RRKM/ME simulations (BF = 0.26 vs an experimentally derived value of 0.48 ± 0.15) (see Table 2). It should be noted that following the electrophilic O atom attack on a ring carbon atom on the lowest A′ triplet PES through the lowest entrance barrier of 3.8 kcal/mol, the initially formed triplet diradical ^3^W1(A′) preferentially undergoes H elimination via an exit barrier (^3^TS2) to produce C_6_H_5_O + H (Figure 1). In contrast, following the electrophilic O atom attack at a ring carbon atom on the first excited ^3^A″ PES through a slightly higher entrance barrier of 4.5 kcal/mol, the initially formed triplet diradical ^3^W1(A″) preferentially and readily undergoes ISC onto the ^1^A′ singlet surface (Figure 1). As discussed in section 4 (also see ref (34)), there are two MECPs between ^3^A″ and ^1^A′. One of them (labeled ISC1) is located at −7.8 kcal/mol with respect to reactants, which is 0.8 kcal/mol above the ^3^W1(A″) state, while the other one (labeled ISC2) is located at −9.2 kcal/mol, which is 0.6 kcal/mol below the ^3^W1(A″) state. ISC is fast at these two MECPs, leading to the singlet state of the adduct followed by an almost barrierless isomerization to benzene oxide (^1^W7), which is located at −54.5 kcal/mol with respect to reactants (see Figure 1). Once formed, benzene oxide can undergo various isomerizations and dissociate to give various products that have been detected experimentally. Specifically, ^1^W7 isomerizes first via a barrier of 43.4 kcal/mol (^1^TS12) to the more stable 2,4-cyclohexadienone (^1^W2) isomer (−81.5 kcal/mol). Subsequently, 2,4-cyclohexadienone can undergo C–H bond cleavage to form phenoxy + H (located at −13.1 kcal/mol with respect to reactants) or competitively isomerize to ^1^W3 (−42.1 kcal/mol) and then ^1^W4 (−48.8 kcal/mol), which ultimately decomposes via ^1^TS4 (−24.2 kcal/mol) to cyclopentadiene + CO, the most exothermic product channel, located at −73.6 kcal/mol with respect to reactants (see Figure 1). ^1^W2 can also isomerize to phenol (^1^W1) through a higher barrier (^1^TS1) located at −30.6 kcal/mol. Phenol in turn can barrierlessly decompose to give phenoxy + H, which is favored with respect to the competitive decomposition to benzyne (C_6_H_4_) + H_2_O (see Figure 1 in ref (34)).

It is appropriate here to comment on the nature of the coproduct of the spin-forbidden CO-forming channel. Clearly, the early suggestion^37^ that the coproduct of CO is the open chain hydrocarbon 3-penten-1-yne was erroneous, as demonstrated by the present and previous theoretical work,^24,48,51^ by the direct observation of cyclopentadiene in the experiment by Taatjes et al.^24^ through accurate measurements of the ionization efficiency curve of the product, and also by the present and previous^10,51^ CMB studies.

It is interesting to examine the BFs of decomposition of the initial singlet intermediate benzene oxide (^1^W7) to C_5_H_6_ + CO and C_6_H_5_O + H in the case of the non-adiabatic reaction of O(^3^P) and the adiabatic reaction of O(^1^D) from the present CMB experiments. As shown in Table 2, for the O(^3^P) reaction via ISC the experimental BF of the C_5_H_6_ + CO channel (0.32 ± 0.14) is nearly twice that of the C_6_H_5_O + H channel (0.18 ± 0.09), while for the O(^1^D) reaction the BF of the C_5_H_6_ + CO and C_5_H_5_ + H + CO channels together (0.34 + 0.62 = 0.96) is nearly 25 times larger than that of the C_6_H_5_O + H channel (0.04). Clearly, the much more internally excited cyloehexadienone (^1^W2) formed in the much more exothermic O(^1^D) reaction preferentially isomerizes to intermediate ^1^W4 rather than dissociating to phenoxy + H, and ^1^W4 leads readily to C_5_H_6_ + CO products via ^1^TS4 (see Figure 1).

We have not attempted a statistical estimate of the product BFs for the O(^1^D) reaction. The two main reasons are (i) given the high entrance energy in the singlet wells, a statistical treatment is not warranted because the reaction dynamics is expected to be dominated by nonstatistical effects, and (ii) it is difficult to calculate individual channel rates because of secondary decompositions. As a matter of fact, although Chen et al.^51^ in their CMB study of the O(^1^D) + C_6_H_6_ reaction did perform statistical calculations of individual microcanonical rate coefficients and also of individual product microcanonical BFs for the reaction as functions of energy (from 5 to 40 kcal/mol), they could not compare the experimentally estimated BFs with the statistically computed BFs at *E*_c_ = 10 kcal/mol because of the secondary dissociations.

### 5.2 Product Recoil Energies and PESs

In discussing the product energy releases we will refer again to the features of the triplet and singlet PESs. Following the O(^3^P) attack on the aromatic ring via the lowest energy barrier of 3.8 kcal/mol (^3^TS1 in Figure 1), the diradical triplet intermediate ^3^W1(A′) (located at −12.8 kcal/mol) is formed. Because of its high energy content, it will decompose to phenoxy + H via ^3^TS2 located at −0.9 kcal/mol with respect to reactants (at 13.3 kcal/mol above the products). The *P*(*E*_T_^′^) for the phenoxy + H products so formed on the triplet PES peaks far away from zero, at 6.4 kcal/mol (see Figure 6b, top panel), which is expected for a nearly direct reaction because of the presence of an exit barrier of about 13 kcal/mol with respect to products (see Figure 1). The *P*(*E*_T_^′^) extends up to the limit of energy conservation (23 ± 3 kcal/mol), and this is consistent with the experimental reaction exothermicity of 14.4 kcal/mol and *E*_c_ = 8.2 kcal/mol. The average fraction of total available energy (*E*_tot_ = *E*_c_ – Δ*H*_0_^0^ = 8.2 + 14.4 = 22.6 kcal/mol) released in translation is ⟨*f*_T_⟩ = 0.34, indicating that the phenoxy radical is highly internally excited (⟨*f*_int_⟩ = 0.66). In contrast, the *P*(*E*_T_^′^) of the phenoxy + H products formed via ISC (the ratio of BFs (phenoxy from triplet)/(phenoxy from singlet) is 0.48/0.18 = 2.7; see Table 2) peaks at an energy closer to zero (about 3 kcal/mol) and dies off at only about 10 kcal/mol, reflecting a significantly smaller fraction of product energy in translation (⟨*f*_T_⟩ = 0.17). The peaking of *P*(*E*_T_^′^) close to zero is typical of reactions proceeding via a long-lived complex mechanism with a statistical product energy distribution. Notably, the *P*(*E*_T_^′^) of phenoxy + H from the O(^1^D) reaction, which evolves on the singlet PES, exhibits similar features, i.e., it also peaks at very low energy (3.5 kcal/mol) but extends up to about 40 kcal/mol, consistent with the larger exothermicity of the O(^1^D) reaction; the average fraction of total available energy in translation (⟨*f*_T_⟩ = 0.16) indicates that the phenoxy radical from O(^1^D) is highly internally excited (⟨*f*_int_⟩ = 0.84). It should be noted that overall the average internal energy of phenoxy from the O(^1^D) reaction (about 50 kcal/mol) is much higher than that for the phenoxy from O(^3^P) (about 6.5 kcal/mol), which is mostly formed adiabatically on the triplet PES (see above). This is the reason why phenoxy from O(^1^D) fragments in the ionizer to *m*/*z* = 65 much more consistently than phenoxy from O(^3^P) does, as already discussed.

With regard to the channel forming C_5_H_6_ + CO from O(^3^P) via ISC, the best-fit *P*(*E*_T_^′^) peaks at about 5 kcal/mol and dies off at about 20 kcal/mol, reflecting a very small fraction of total available energy in product translation (⟨*f*_T_⟩ = 0.08) (Figure 6b, fourth panel from the top). This indicates that the two molecular products are very highly internally excited. In contrast, the *P*(*E*_T_^′^) for the same products formed from the O(^1^D) reaction peaks at about 26 kcal/mol and extends up to about 90 kcal/mol, reflecting a significantly larger fraction of energy in product translation (⟨*f*_T_⟩ = 0.27) (Figure 6b, fifth panel from the top). This larger fraction of energy in translation indicates that a significant fraction of the internal (electronic) energy of excited atomic oxygen is converted into translational energy of the products. Interestingly, 73% of the total available energy (*E*_tot_ ≈ 128 kcal/mol) residing in internal excitation of the CO + C_5_H_6_ products is large enough that a fraction of internally excited cyclopentadiene can unimolecularly readily fragment to C_5_H_5_ + H (see Figure 1). Indeed, the experimental data indicate that this is actually the dominant product channel, with a BF of 0.62 ± 0.15 (Table 2), to be compared with the BFs of 0.34 ± 0.10 and 0.04 ± 0.02 for the C_5_H_6_ + CO and C_6_H_5_O + H channels, respectively, from O(^1^D). This result is in agreement with the findings of Chen et al.^51^ at *E*_c_ = 10 kcal/mol, according to which the three-body channel is dominant while the phenoxy and CO channels are minor.

The total available energy for the three-body channel is about 47 kcal/mol. The best-fit *P*(*E*_T_^′^), derived as described in section 3.3, peaks at about 5 kcal/mol and extends up to about 36 kcal/mol, corresponding to a sizable fraction of the total available energy released as product translational energy (⟨*f*_T_⟩ ≈ 0.24). As can be seen from Figure 6b, the *P*(*E*_T_^′^) distributions for C_5_H_6_ + CO (channel 5) and C_5_H_5_ + H + CO (channel 6) are very different. In fact, the peak of the cyclopentadienyl radical from channel 6 occurs at about 220 μs in the TOF at *m*/*z* = 65, while that of cyclopentadiene from channel 5 occurs at about 140 μs (see Figure 5b and Figure S1).

The mechanism of C_5_H_6_ + CO formation sees the bridge addition of O(^1^D) to two adjacent carbons of the ring to form benzene oxide (^1^W7), which then isomerizes by hydrogen migration to ^1^W2 (2,4-cyclohexadienone). ^1^W2 competitively undergoes C–H bond cleavage to give C_6_H_5_O + H or isomerization to ^1^W3 and finally, via ring contraction, to ^1^W4, which leads to C_5_H_6_ + CO via ^1^TS_4_. Interestingly, C_5_H_6_ is formed with enough internal energy to undergo fast barrierless unimolecular decay to H + C_5_H_5_, forming a three-body reaction pathway (channel 6), which is overall exoergic by about 38 kcal/mol with respect to the O(^1^D) + C_6_H_6_ reactants (Figure 1).

### 5.3 Product Branching Fractions and Extent of ISC

As mentioned above, the experimentally derived overall branching fractions for the six detected competing channels 1–6 from the overall O(^3^P, ^1^D) + C_6_H_6_ reactions are reported in Table 1. A few important aspects to note from this table are the following: (i) under our experimental conditions, about 95% of the total reactive yield is due to the O(^1^D) reactions, with the overall reactive yield from O(^3^P) being only about 5%; (ii) the dominant pathway is the three-body channel (channel 6) originating from the O(^1^D) reaction, followed by channel 5, corresponding to the formation of C_5_H_6_ + CO from O(^1^D); (iii) the other four possible channels are all minor yet non-negligible; (iv) the total yield of phenoxy + H from O(^3^P) (BF = 0.032 ± 0.011) is very similar to that from O(^1^D) (BF = 0.035 ± 0.010); (v) the C_5_H_6_ + CO channel from O(^3^P) (BF = 0.015 ± 0.007) amounts to only about 5% of that from O(^1^D) (BF = 0.32 ± 0.09); (vi) a small quantity of phenol is observed from the O(^3^P) reaction (channel 3).

As already mentioned, the individual BFs for the O(^3^P) and O(^1^D) reactions are derived from Table 1 and listed in Table 2. There, the experimental BFs for the O(^3^P) reaction channels are compared with our statistical predictions on the coupled triplet and singlet PESs, including ISC. As can be seen from Table 2, the experimental and simulation results are in reasonable agreement when it comes to total formation of phenoxy + H (BF_RRKM/ME_ = 0.41 vs BF_CMB_ = 0.66 ± 0.24), with the direct fraction of phenoxy + H on the triplet PES being theoretically underestimated (0.26 vs 0.48 ± 0.15). However, theory somewhat overestimates the experimental C_5_H_6_ + CO channel BF (0.59 vs 0.32 ± 0.14). Of course, theory under single-collision conditions predicts zero phenol, while experimentally we derived a BF of 0.02 ± 0.01 for phenol surviving all the way up to the detector. When we sum all of the triplet product yields and all of the singlet product yields for the various channels of the O(^3^P) reaction, we find that the extent of ISC is experimentally 0.52 ± 0.15 against a theoretical prediction of 0.74, which highlights a reasonable agreement between experiment and theory.

Table 2 also reports BFs derived from the kinetic study of the O(^3^P) + benzene reaction at 900 K and 4 Torr by Taatjes et al.^24^ along with the results of our statistical simulations carried out for the same kinetic experimental conditions. Notably, the present theory somewhat overestimates the phenoxy + H channel (0.59 vs 0.33 ± 0.13) and underestimates the C_5_H_6_ + CO channel (0.14 vs 0.33 ± 0.08) but provides a good estimate of the phenol formation channel (0.27 vs 0.33 ± 0.08). Those values correspond to an ISC extent of 0.67 ± 0.16 from the kinetic experiment at 900 K and 4 Torr, in rather satisfactory agreement with the value of 0.41 returned by theory, which, assuming a theoretical uncertainty comparable to the experimental one (±25%), would fall within the lower experimental error bound.

It is interesting to examine the variation of the ISC with temperature for the O(^3^P) + benzene reaction. The variations of the BFs and of the extent of ISC with temperature (in the range 300–900 K) were previously reported in Figure 4a,b of ref (34) and are discussed there.

### 5.4 Dynamics of the O(^1^D) + C_6_H_6_ Reaction

As can be seen from the singlet PES (blue curves in Figure 1), the best-fit CM functions reported in Figure 6, and the BFs reported in Table 2, the O(^1^D) + C_6_H_6_ reaction starts with the barrierless addition of O(^1^D) to two adjacent carbons of the benzene ring to form benzene oxide (^1^W7), which via various isomerizations can lead dominantly to C_5_H_6_ + CO and C_6_H_5_O + H, with a large part of the former products having enough internal excitation to undergo secondary dissociation of cyclopentadiene to cyclopentadienyl (C_5_H_5_) + H (three-body channel, channel 6). The major channel is the formation of the three fragments C_5_H_5_ + H + CO (BF = 0.62 ± 0.15), with the channel forming C_5_H_6_ + CO (channel 5) being about half (BF = 0.34 ± 0.10) of the three-body channel and the channel forming C_6_H_5_O + H (channel 4) being minor (BF = 0.04 ± 0.02) (see Table 2).

We note that, on the one hand, our experimental results on O(^1^D) + benzene are very similar to those reported by Chen et al.:^51^ both studies found that the three-body channel is the dominant one. On the other hand, in the pulsed CMB experiment of Chen et al.,^51^ only TOF spectra of the products were measured at selected LAB angles, while in our continuous CMB experiments we were also able to directly measure with high accuracy the total LAB angular distribution for each product mass and then the product TOF spectra at selected LAB angles. We remind the reader that the area of a TOF spectrum at a given LAB scattering angle corresponds to the intensity of the LAB angular distribution at that given angle. The availability of the angular distribution with a fine angular grid of data points provides more accurate information and facilitates the data analysis and the derivation of the CM functions (i.e., of the reaction dynamics). Deviations of the present results from previous work^51^ concerning the relative importance of the H-forming channel (channel 4) and the CO-forming channel (channel 5) with respect to the three-body channel (channel 6) were discussed earlier in section 3.4.

## 6 Conclusions

Although in the past decades the O(^3^P) + benzene reaction was extensively studied from both theoretical and experimental points of view because of its relevance in fuel combustion, the characterization of its detailed mechanism and dynamics, such as the primary product distribution and the role of ISC, remained to be done. In the present work, the O(^3^P) + benzene reaction dynamics was investigated experimentally by the CMB scattering method with MS detection and TOF analysis at *E*_c_ = 8.2 kcal/mol, and the primary products and their branching fractions were determined. The experimental results were analyzed with the support of synergistic high-level quantum-chemical calculations of the underlying triplet and singlet PESs and statistical (RRKM/ME) simulations on these PESs with non-adiabatic effects (i.e., ISC) taken into account in order to gain a deeper and more comprehensive understanding of the reaction mechanism and dynamics. This combined experimental/theoretical study on the O(^3^P) + benzene benchmark system extends to aromatic hydrocarbons our recent combined experimental/theoretical studies^19−23,29,32−34,77^ on O(^3^P) + C_2_, C_3_, and C_4_ unsaturated hydrocarbons and can serve as a gateway to more complex chemical pathways available in larger aliphatic/aromatic hydrocarbons.

Notably, although under our experimental conditions the concentration of O(^1^D) in the oxygen beam is ≤10%, its reactivity with benzene at *E*_c_ = 8.2 kcal/mol, as expected because the O(^1^D) + benzene reaction is barrierless, appears to be much higher (by about 2 orders of magnitude) than that observed for O(^3^P) (whose reaction has an entrance barrier of 3.8–4.5 kcal/mol), in agreement with Chen et al.^51^ and with the rate constants of the O(^3^P) reaction determined in previous kinetics studies.^40−46^ The detailed dynamics of the O(^1^D) + benzene reaction determined in this study is in agreement with the results of the previous detailed pulsed CMB study of Chen et al.^51^ In particular, the three-body channel leading to cyclopentadienyl + H + CO is assessed in both studies to be the dominant product channel (BF > 0.6), while the cyclopentadiene + CO and phenoxy + H channels serve a minor role.

The derived reaction mechanism of the O(^3^P) + benzene reaction involves the initial electrophilic attack of the O atom on the π system of the aromatic ring (on a C atom) on the two lowest triplet T1(^3^A′) and T2(^3^A″) PESs, with the formation of the triplet diradical adducts ^3^W1(A′) and ^3^W1(A″). These adducts, under single-collision conditions, can undergo competitive C–H bond cleavage on the lowest triplet T1(^3^A′) PES and intersystem crossing (ISC1 and ISC2 at MECP1 and MECP2, respectively) from the excited T2(^3^A″) PES to form benzene oxide. The latter readily isomerizes to 2,4-cyclohexadienone, which in turn can competitively dissociate to give C_6_H_5_O + H and, via two successive isomerization steps involving ring contraction, C_5_H_6_ + CO. Because of the very long lifetime of the 2,4-cyclohexadienone and isomeric phenol intermediates, a small fraction of these adducts actually survive long enough (≥300 μs) to reach the mass spectrometer detector.

We have characterized the dynamics (center-of-mass product angular and translational energy distributions) of the main open reaction channels, namely, those leading to (in order of decreasing importance and with branching fractions in parentheses) C_6_H_5_O + H (0.66 ± 0.24, of which 0.48 ± 0.15 is from the triplet PES and 0.18 ± 0.09 is from the singlet PES via ISC), C_5_H_6_ + CO (0.32 ± 0.14), and phenol (0.02 ± 0.01). Therefore, under single-collision conditions at *E*_c_ = 8.2 kcal/mol, the reactive interaction of O(^3^P) with benzene mainly produces the radical channel phenoxy + H (overall BF = 0.66) but can also break apart the aromatic ring to produce significant amounts of cyclopentadiene + CO (BF = 0.32). A small fraction of the adduct is also observed. Because some of the observed products can be formed only via ISC from the triplet PES to the singlet PES, we have inferred the extent of ISC from the product branching fractions. Our data indeed suggest that ISC is extremely relevant, accounting alone for 52 ± 15% of the product yield at the experimental *E*_c_. It should be noted that this value is comparable to that observed in the O(^3^P) + 1-butene reaction at a comparable *E*_c_ (50 ± 15%).^22^ As summarized in Table 2, the experimental and theoretical BFs determined in this work are in reasonable agreement with each other, as are the extents of ISC (experimentally 52 ± 15% and theoretically 74%). Significant differences between experimental and statistical BFs are mainly limited to the H displacement channel occurring adiabatically on the triplet PES, a process known to be not fully statistical. The impact of the model parameters on the theoretically predicted BFs has been examined.

Comparison of the theoretically predicted BFs at 900 K and 4 Torr with those at *E*_c_ = 8.2 kcal/mol has provided useful information on the variation of BFs with collision energy (temperature).^34^ Notably, the overall predicted yield of product channels from the singlet PES (about 52% at *E*_c_ = 8.2 kcal/mol) remains essentially the same (54%) at 900 K and 4 Torr. However, under those thermal conditions the calculated fraction of stabilized product increases strongly (from BF = 0.02 under the CMB conditions to BF = 0.27 under the thermal conditions), while the fractions of C_5_H_6_ + CO and of C_6_H_5_O + H decrease (0.14 vs 0.32 and 0.13 vs 0.18, respectively). Clearly, while the extents of ISC under the two sets of experimental conditions (CMB at *E*_c_ = 8.2 kcal/mol and kinetics at 900 K and 4 Torr) are comparable, in the thermal case third-body stabilization plays a crucial role because of the multiple-collision environment. However, as shown in Table 2, it is noteworthy that the statistical calculations at 900 K and 4 Torr overestimate considerably (by nearly a factor of 2) the overall amount of the radical channel (phenoxy + H) from the kinetic experiment (BF = 0.59 theory vs 0.33 experiment) but underestimate by a similar amount the fraction of the molecular channel cyclopentadiene + CO (BF = 0.14 vs 0.33). Most notably, the amounts of the spin-forbidden molecular C_5_H_6_ + CO channel are comparable at *E*_c_ = 8.2 kcal/mol (BF = 0.32 ± 0.14) and at 900 K and 4 Torr (BF = 0.33 ± 0.08).

One of the main results of this work is that the CMB BFs measured in the present work and those determined in kinetic experiments^24^ cannot be reproduced using the same statistical model, even if some of the model parameters are modified within their uncertainty ranges. This disagreement may be determined by different aspects, such as secondary chemistry contributing to the system reactivity in the kinetic experiments or dynamic effects not caught by the statistical model influencing the CMB dynamics. It is our opinion that this shortcoming may be addressed in the future by performing new kinetic experiments (or reinterpreting the existing ones^24^ using appropriate reactor and kinetic models) and performing simulations of the CMB system using molecular dynamics approaches.

In the context of combustion processes, the most interesting and important result produced by this combined experimental and theoretical study of the complex mechanism of the O(^3^P) + benzene reaction is that once the theoretical statistical approach and description were reasonably validated by a satisfactory and encouraging comparison with the CMB experimental results, theory could be used to generate channel-specific rate constants as a function of temperature and pressure over a wide *T* range (from 300 to 2200 K) and *p* range (from 0.1 to 100 atm) (see Table 4). We expect the valuable insights provided by these channel-specific rate constants to significantly support and expedite a much-needed improvement of current hydrocarbon combustion models.

## Supporting Information Available

Previous experimental and theoretical studies on the O(^3^P, ^1^D) + benzene reactions, discussion of the abstraction channel in the O(^3^P) + C_6_H_6_ reaction forming OH + C_6_H_5_ (phenyl), and sensitivity of TOF distributions to C_5_H_6_ (cyclopentadiene) and C_5_H_5_ (cyclopentadienyl) formation from O(^1^D) (PDF)
