# Supplementary material for: Crossed-Beam and Theoretical Studies of the O(3P, 1D) + Benzene Reactions: Primary Products, Branching Fractions, and Role of Intersystem Crossing
Source: J Phys Chem A. 2021 Sep 17;125(38):8434–53. doi: 10.1021/acs.jpca.1c06913 (PMC8488941; doi:10.1021/acs.jpca.1c06913)
Supplement: Supplementary file 1 — jp1c06913_si_001.pdf [file jp1c06913_si_001.pdf]

Electronic Supporting Information for the paper

Crossed-Beams and Theoretical Studies of the O(^{3}P, ^{1}D) + Benzene Reactions: Primary Products, Branching Fractions, and Role of Intersystem Crossing

Gianmarco Vanuzzo, Adriana Caracciolo, Timothy K. Minton[§,#], Nadia Balucani*, Piergiorgio Casavecchia*

Dipartimento di Chimica, Biologia e Biotecnologie, Università degli Studi di Perugia, 06123 Perugia, Italy

Carlo de Falco

MOX – Modellistica e Calcolo Scientifico, Dipartimento di Matematica, Politecnico di Milano, 20133 Milano, Italy

Alberto Baggioli, Carlo Cavallotti*

Dipartimento di Chimica, Materiali e Ingegneria Chimica, Politecnico di Milano, 20131 Milano, Italy

Carlo Cavallotti[1*], Carlo De Falco[2], Luna Pratali Maffei[1], Adriana Caracciolo[3], Gianmarco Vanuzzo[3], Nadia Balucani[3], and Piergiorgio Casavecchia[3*]

This file contains:

S1. Previous experimental and theoretical studies on the O(^{3}P, ^{1}D) + benzene reactions.

S2. On the abstraction channel in the O(^{3}P) + C_6H_6 reaction forming OH + C_6H_5 (phenyl).

S3. Sensitivity of time-of-flight (TOF) distributions to C_5H_6 (cyclopentadiene) and C_5H_5 (cyclopentadienyl) formation from O(^{1}D).

S1. Previous experimental and theoretical studies on the O(^{3}P, ^{1}D) + benzene reactions.

Among the earliest investigations on the O(^{3}P) + benzene reaction, we note the kinetics experiment carried out by Cvetanović and coworkers in 1961 at room temperature, where oxygen atoms were produced by mercury photosensitized decomposition of nitrous oxide.[1] These measurements indicated that the main reaction product is a non-volatile material, or rather an opaque yellow film, whose quantity was accurately measured by its combustion and subsequent measurement of evolved CO_2. In addition to this substance, Cvetanović observed a small amount of phenol and carbon monoxide. Interestingly, in this study it was found that the O(^{3}P) reactivity with benzene is much slower than that observed in analogous experiments with olefins.[2] Subsequently, numerous kinetics studies were performed over the years on the O(^{3}P) + benzene reaction aimed at determining the rate constant as a function of temperature.[3-11]

In 1977, Sloane[12] studied in "near" collision-free conditions the O(^{3}P) + benzene reaction using thermal beams of the reactants and a fixed mass spectrometer detector with tunable electron energy. In this study, the phenol (adduct) and the CO + C_5H_6 reaction channel were detected, thus corroborating the previous kinetics studies;[1] however, the H-displacement channel leading to phenoxy formation was not observed. The co-product accompanying carbon monoxide could be either cyclopentadiene or an open-chain hydrocarbon. To verify this hypothesis, Sloane compared the ionization potentials of the possible products and concluded that the observed product at mass 66 was likely the open chain olefin 3-penten-1-yne.[12] In 1980, the dynamics of the O(^{3}P) + benzene reaction were investigated by Sibener *et al.*[13] at several collision energies (E_c = 2.5, 6.4, and 8.5 kcal/mol) by the crossed molecular beam (CMB) technique with rotatable mass spectrometer (MS) detector and time-of-flight (TOF) analysis. In this study, it was observed that the O(^{3}P) + C_6H_6 reaction mainly leads to H + C_6H_5O (phenoxy), while CO + C_5H_6

(likely cyclopentadiene) was concluded to be a minor channel (≤5%). In addition, the detection at the parent mass *m/z* = 94 of the reaction adduct (C_6H_6O) suggested that the lifetime of the phenol intermediate (or part of it), formed via ISC, is longer than its travel time (about 300 μs) from the collision region to the MS detector. In 1986 the O(^{3}P) + benzene reaction was also studied by the CMB method by Grice *et al.*,[14] but only the phenoxy channel was observed.

From a theoretical point of view, in 2007, the lowest lying triplet and singlet PESs for the O(^{3}P) + benzene reaction were characterized by Nguyen *et al.*[15] by using a complete basis set CBS-QB3 level of theory, and the primary products distribution for each of these surfaces was estimated by applying RRKM/ME calculations. This study superseded the earlier theoretical investigation of Hodgson *et al.*[16] Nguyen *et al.* concluded that the O(^{3}P) + benzene reaction mainly occurs via an O-addition mechanism on the triplet PES leading to C_6H_5O (phenoxy) + H, together with some phenol and/or benzene oxide/oxepin (adduct). While the atomic hydrogen displacement channel was exclusively derived from the triplet PES, it was concluded that the phenol adduct was formed on the singlet PES via intersystem crossing (ISC); however, ISC effects were not included in the calculations. Three years later (2010), Taatjes *et al.*[17] identified for O(^{3}P) + benzene as primary products phenol, phenoxy, and cyclopentadiene using multiplexed photoionization mass spectrometric chemical kinetics experiments with tunable VUV synchrotron radiation. Furthermore, it was observed that the branching fractions (BFs) of the primary products are significantly temperature and pressure dependent (in the 300-900 K and 1-10 torr ranges, respectively). At 300 K the phenol, in fact, appeared to be dominant, but the BFs for the three channels became comparable (BF=0.33) with the increase of temperature to 900 K. [17]

In contrast to $O(^3P)$ + benzene, there have been no reports on the dynamics of the reaction $O(^1D)$ + benzene until 2008, when Chen *et al.*[18] published a very detailed study of $O(^1D) + C_6H_6$ and $O(^1D) + C_6D_6$ in pulsed CMB experiments with MS detection and TOF analysis, in which product translational energy distributions and product branching ratios were determined at the E_c of 10 kcal/mol. The CMB study was complemented by measurements of internal-state distributions and branching ratios of CO and OH products with time-resolved Fourier-transform infrared (FTIR) emission in a flow system, and by electronic structure calculations at the G2M(CC5) level of theory to predict the energetics of the reaction intermediates and transition states of the singlet PES and perform statistical calculations of rates, rate coefficients, and branching ratios. Notably, in that instance the atomic oxygen beam was generated by pulsed laser photolysis at 157.6 nm of a pulsed molecular O_2 beam, which produces $O(^3P)$ and $O(^1D)$ species in equal fractions. The experimental data were corrected for the $O(^3P)$ contributions by conducting an experiment using a pure $O(^3P)$ beam (obtained by NO_2 photolysis at 308 nm) having a similar velocity. In that study, the previous CMB experimental observations[13] of phenol (minor), phenoxy (main product) and cyclopentadiene (minor product) were confirmed; however, the data of the $O(^3P)$ study were not reported, while only the $O(^1D)$ distributions, corrected for the $O(^3P)$ contributions, were given. It was found that, for $O(^1D)$ + benzene, the 3-body channel H + CO + C_5H_5 (cyclopentadienyl radical) is dominant, while the radical channel H + phenoxy, the molecular channel CO + cyclopentadiene, and the radical channel OH + phenyl, were concluded to be minor. Notably, contrary to what was observed with $O(^3P)$, phenol was not detected as an adduct of $O(^1D)$ + benzene. The product center-of-mass (CM) angular and translational energy distributions were derived for all observed reactive channels. Those experimental data for the $O(^1D)+C_6H_6$ reaction at E_c=10.0 kcal/mol and the derived CM

functions are discussed in the main text in relation to the present results for the same reaction, obtained at the somewhat lower E_c of 8.2 kcal/mol.

In summary, despite the numerous previous studies from both theoretical and experimental points of view, the detailed dynamics of the $O(^3P)$ + benzene reaction appears to be still in question. In particular, the extent of the spin-forbidden pathway leading to CO + C_5H_6 products under single collision conditions, as well as the lifetime of the triplet intermediate that leads to the H + phenoxy channel, and the detailed dynamics of this channel are still uncertain. Specifically, there appears to be a significant disagreement between the BFs derived from CMB studies[13,19] and those recently obtained from kinetic investigations with synchrotron radiation.[17] We note that the formation of phenol (channel (3) in main text) or cyclopentadiene + CO (channel (2) in main text) leads to free-radical chain termination during benzene oxidation at high temperatures; in contrast, the production of phenoxy radical + H (channel (1) in main text) provides secondary chain branching. We can therefore expect significant effects of the product BFs on models of benzene oxidation.

S2. On the abstraction channel in the $O(^3P)$ + C_6H_6 reaction forming OH + C_6H_5 (phenyl).

Notably, in the present CMB experiments at E_c=8.2 kcal/mol we have not observed reactive signal at *m/z* = 17 which rules out (within our sensitivity) the H abstraction pathway leading to OH formation. Indeed, H abstraction is not expected to contribute at the experimental E_c for the $O(^3P)$ reaction[15,18] because it is significantly endothermic (by about 12 kcal).[15] However, some OH could arise from the $O(^1D)$ reaction, but this would be difficult to detect, because of several factors: unfavorable kinematics, low concentration of $O(^1D)$ in the O-beam, presence of ^{17}O natural isotopic abundance, and high inherent detector background at *m/z*=17 (due to water

dissociative ionization). Notably, to partially overcome these difficulties, Chen *et al.*[18] performed a CMB experiment at E_c=12 kcal/mol on the isotopically labeled reaction $^{18}O(^1D)$ + C_6D_6 and observed a weak signal at *m/z*=20 (^{18}OD) within a limited range of LAB angles. From this, formation of ^{18}OD was estimated to be minor, with a branching fraction of <0.1. In our study at E_c=8.2 kcal/mol we assume the H abstraction channel to be minor and neglect it in the estimate of the branching fractions.

S3. Sensitivity of time-of-flight (TOF) distributions to C_5H_6 (cyclopentadiene) and C_5H_5 (cyclopentadienyl) formation from $O(^1D)$ + C_6H_6.

It may be enlightening to look at Figure SI-1 which compares the TOF spectra at Θ=28° for *m/z* = 66 and 65 for the $O(^3P, ^1D)$ + C_6H_6 reactions at E_c=8.2 kcal/mol. Together with the TOF spectra at various angles for the same two masses, shown in the Figures 4b and 5b of the main text, Figure SI-1 illustrates the relative time scales of the contributions of the various reaction channels of the $O(^3P,^1D)$ + benzene reactions at E_c=8.2 kcal/mol. In Figure SI-1 vertical lines indicate the position of the peak of the various indicated contributing products. Note that cyclopentadienyl (C_5H_5) from the 3-body channel from $O(^1D)$ can be clearly seen at its parent mass (*m/z*=65) (bottom panel). Note also that the very fast cyclopentadiene (C_5H_6) product from $O(^1D)$ can be seen very clearly at its parent mass (*m/z*=66) (top panel), while at its daughter ion mass of *m/z*=65 it appears only as a fast shoulder on the intense peak of cyclopentadienyl from $O(^1D)$. Although the BF of cyclopentadiene from $O(^3P)$ is similar to that of cyclopentadiene from $O(^1D)$ (see Table 2 in main text), its peak is much less intense (see top panel) than that of cyclopentadiene from $O(^1D)$ because $O(^1D)$ is much more reactive than $O(^3P)$ (see main text). Finally, note that phenoxy from $O(^1D)$ and phenoxy from $O(^3P)$ on the triplet PES (which is

strongly forward scattered, as shown in Figure 6 of the main text) are well visible at their daughter ion *m/z*=65 (bottom panel), while phenoxy from O(^{3}P) via ISC (which has a significantly smaller BF with respect to the phenoxy from the other two pathways (see Table 1 in main text)) is so narrowly confined around the CM angle (48°) that it has almost completely died off at Θ=28° (while it can be seen at angles closer to the CM angle - see Figure 5 in main text).

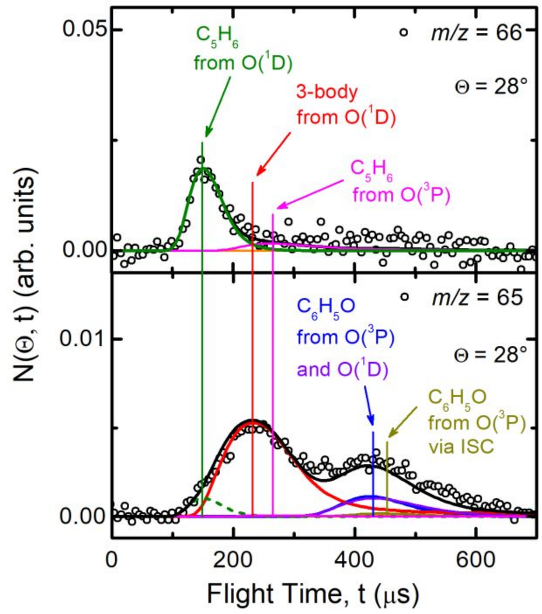

Figure S1. Comparison of the TOF spectra for *m/z*=66 (top panel) and *m/z*=65 (bottom panel) at the same LAB angle Θ=28° for the O(^{3}P, ^{1}D) + benzene reactions at E_c=8.2 kcal/mol.

REFERENCES

(1) Boocock, G.; Cvetanović, R. J. Reaction of Oxygen Atoms with Benzene. *Can. J. Chem.* **1961,** *39*, 2436-2443.

(2) Cvetanović, R. J. Evaluated Chemical Kinetic Data for the Reactions of Atomic Oxygen O(^{3}P) with Unsaturated Hydrocarbons. *J. Phys.Chem. Ref. Data* **1987,** *16*, 261.

(3) Mani, I. ; Sauer, M. C. Jr. A Pulsed-Radiolysis Study of the Gas-Phase Reaction of Oxygen Atoms with Benzene and Related Compounds: Rate Constants and Transient Spectra *Adv. Chem. Ser.* **1968**, *82*, 142-152.

(4) Bonanno, R. A.; Kim, P.; Lee, J. H.; Timmons, R. B. Kinetics of the Reaction of O(^{3}P) Atoms with Benzene. *J. Chem. Phys.* **1972**, *57*, 1377.

(5) Atkinson, R.; Pitts, J. N., Absolute Rate Constants for the Reaction of O(^{3}P) Atoms with Selected Alkanes, Alkenes, and Aromatics as Determined by a Modulation Technique. *J. Phys. Chem.* **1974**, *78*, 1780-1784.

(6) Colussi, A. J.; Singleton, D. L.; Irwin, R. S.; Cvetanovic, R. J. Absolute Rates of Oxygen(^{3}P) Atom Reactions with Benzene and Toluene *J. Phys. Chem.* **1975**, *79*, 1900-1903.

(7) Atkinson, R.; Pitts, J. N. Rate Constants for the Reaction of O(^{3}P) Atoms with Benzene and Toluene over the Temperature Range 299–440 K. *Chem. Phys. Lett.* **1979**, *63*, 485-489.

(8) Nicovich, J. M.; Gump, C. A.; Ravishankara, A. R. Rates of Reactions of O(^{3}P) with Benzene and Toluene. *J. Phys. Chem.* **1982**, *86*, 1684-1690.

(9) Leidreiter, H. I.; Wagner, H. Gg. An Investigation of the Reaction between O(^{3}P) and Benzene at High Temperatures. *Z. Phys. Chem.* **1989**, *165*, 1-7.

(10) Tappe, M.; Schliephake, V.; Wagner, H. Gg. Reactions of Benzene, Toluene and Ethylbenzene with Atomic Oxygen O(^{3}P) in the Gas Phase *Z. Phys. Chem.* **1989**, *162*, 129-145.

(11) Ko, T.; Adusei, G. Y.; Fontijn, A. Kinetics of the O(^{3}P)+C_6H_6 Reaction over a Wide Temperature Range. *J. Phys. Chem.* **1991**, *95*, 8745-8748.

(12) Sloane, T. M. Reaction Product Identification from O(^{3}P)+Benzene, Toluene, and 1,3,5-Trimethylbenzene Collisions in Crossed Molecular Beams. *J. Chem. Phys.* **1977,** *67*, 2267-2274.

(13) Sibener, S. J. Buss, R. J.; Casavecchia, P.; Hirooka T.; Lee, Y. T. A Crossed Molecular Beams Investigation of the Reactions O(^{3}P)+C_6H_6, C_6D_6. *J. Chem. Phys.* **1980**, *72*, 4341-4349.

(14) Ureña, A. G.; Hoffmann, S. M. A.; Smith, D. J.; Grice, R., Translational Energy Threshold for the Reaction of Oxygen Atoms with Benzene Molecules. *J. Chem. Soc. Faraday Trans. 2: Mol. Chem. Phys.* **1986,** *82*, 1537-1541.

(15) Nguyen, T. L.; Peeters, J.; Vereecken, L., Theoretical Reinvestigation of the O(^{3}P) + C_6H_6 Reaction: Quantum Chemical and Statistical Rate Calculations. *J. Phys. Chem. A* **2007**, *111*, 3836-3849.

(16) Hodgson, D.; Zhang, H.-Y.; Nimlos, M. R.; McKinnon, J. T., Quantum Chemical and RRKM Investigation of the Elementary Channels of the Reaction C_6H_6 + O(^{3}P). *J. Phys. Chem. A* **2001,** *105*, 4316-4327.

(17) Taatjes, C. A.; Osborn, D. L.; Selby, T. M.; Meloni, G.; Trevitt, A. J.; Epifanovsky, E.; Krylov, A. I.; Sirjean, B.; Dames, E.; Wang, H. Products of the Benzene + O(^{3}P) Reaction. *J. Phys. Chem. A* **2010,** *114*, 3355-3370.

(18) Chen, H.-F.; Liang, C.-W.; Lin, J. J.; Lee, Y.-P.; Ogilvie, J. F.; Xu, Z. F.; Lin, M. C. Dynamics of Reactions O(^{1}D)+C_6H_6 and C_6D_6. *J. Chem. Phys.* **2008,** *129*, 174303 (13pp).

(19) Cavallotti, C.; De Falco, C.; Pratali Maffei, L.; Caracciolo, A.; Vanuzzo, G.; Balucani, N.; Casavecchia, P. A Theoretical Study of the Extent of Intersystem Crossing in the O(^{3}P) + C_6H_6 Reaction with Experimental Validation. *J. Phys. Chem. Lett.* **2020**, *11*, 9621-9628.
